# Enhanced initiation of somatic embryos in suspension cultures of *Aesculus flava* and metabolic profile of zygotic embryos and somatic embryos during their development

**DOI:** 10.3389/fpls.2025.1736161

**Published:** 2026-01-14

**Authors:** Snežana Zdravković-Korać, Uroš Gašić, Slađana Jevremović, Branka Uzelac, Maja Belić, Dušica Ćalić, Jelena Milojević

**Affiliations:** Department of Plant Physiology, Institute for Biological Research “Siniša Stanković” - National Institute of the Republic of Serbia, University of Belgrade, Belgrade, Serbia

**Keywords:** *Aesculus flava*, cryopreservation, embryogenic suspension, LC/MS profiling, phenolics, saponins, somatic embryos, zygotic embryos

## Abstract

Zygotic embryos (ZEs) of *Aesculus* species contain a plethora of health-promoting phytochemicals that are used in the pharmaceutical industry and traditional medicine. However, the seed yield decreases due to various stresses, and seeds often contain elevated levels of heavy metals as they are mostly collected from urban environments. Somatic embryos (SEs) could serve as an alternative source of these phytochemicals. Therefore, this study aimed to develop protocols for efficient initiation, regeneration, proliferation, and cryopreservation of *A. flava* SEs, while also establishing a metabolic profile of ZEs and SEs at successive developmental stages. The frequency of initiation of SEs from the filament-derived friable callus was approximately eightfold higher for explants cultured in liquid medium than on solid medium. Embryogenic suspensions with sustained proliferation and high embryogenic capacity were established and maintained efficiently by size fractionation of embryogenic cell aggregates. The selected cell lines were successfully cryopreserved by encapsulation and slow cooling, with 75% recovery from liquid nitrogen. LC/MS characterization of the ethanolic extracts revealed 117 metabolites: benzoic and cinnamic acid derivatives, flavonoids and saponins, including 58 new compounds. This analysis also provided valuable insights into dynamic alterations in specialized metabolites during embryo development. SEs in early developmental stages primarily contained flavonoids, while ZEs mainly contained saponins, whereas cotyledonary-stage SEs (CSEs) contained both flavonoids and saponins along with considerable amounts of flavan-3-ols and procyanidins. Thus, with 102/117 compounds detected, the CSEs obtained from suspension cultures may represent a promising source of metabolites for the food, pharmaceutical, and cosmetic industries. Further optimization of the protocol is required to ensure its robust applicability across *A. flava* clones, together with validation of metabolite yield, purity, and bioactivity.

## 1 Introduction

The genus *Aesculus* comprises 12 species that are widely distributed in the temperate regions of the northern hemisphere (Harris et al., 2009). These species are among the most attractive ornamental trees or shrubs, including a number of varieties, cultivars and interspecific hybrids (Hardin, 1957; dePamphilis and Wyatt, 1989). In addition to their use as ornamental plants, *Aesculus* species are used for traditional and medicinal purposes, as they contain more than 200 valuable, health-promoting phytochemicals (Zhang et al., 2010; Yan et al., 2025), including saponins and flavonoids (Kapusta et al., 2007; Kimura et al., 2017; Zhang et al., 2020; Green et al., 2021; Bielarska et al., 2022; Cruz et al., 2024). These phytochemicals have been found in all studied *Aesculus* species and all plant organs: fruits, seeds, bark, leaves, and flowers (Bombardelli et al., 1996; Zhang et al., 2020; Green et al., 2021; Owczarek et al., 2021; Sun et al., 2023; Dridi et al., 2023; Cruz et al., 2024). Nevertheless, only *A. hippocastanum* and *A. chinensis* have to date been officially recognized for medicinal purposes, likely due to their widespread distribution (Zhang et al., 2010), while other *Aesculus* species are used by local communities for medicine, food, and animal feed (Mohapatra et al., 2024).

Aescin, a mixture of triterpene glycosides, is the most valued phytochemical of *Aesculus* plants, used commercially in various formulations for the treatment of chronic venous insufficiency, hemorrhoids and post-operative oedema (Bombardelli et al., 1996; Gallelli, 2019) and in the cosmetic industry as an anti-cellulite and anti-ageing agent (Wilkinson and Brown, 1999). Aescin has numerous health-promoting effects, (reviewed in Li et al., 2023) and is also recognized as a promising delivery system for macromolecules in biomedicine (Fuchs et al., 2017; Cheong et al., 2018) and in cosmetic and food products, as it can stabilize emulsions and foams and dissolve hydrophobic molecules (Golemanov et al., 2013). Beyond aescin, numerous therapeutically valuable phytochemicals, such as flavonoids, procyanidins, and other phenolic compounds, have been identified in the seeds, leaves, and flowers of *Aesculus* species. These phytochemicals also exhibit numerous health-promoting effects (Chen and Chen, 2013; Kimura et al., 2017; Zhao et al., 2019; Ruwizhi and Aderibigbe, 2020; Green et al., 2021; Cruz et al., 2024) and are consequently proposed as food additives and dietary supplements (Yin et al., 2022). In recent years, there has been a growing demand for plant-derived natural antioxidants in the food industry, as they are non–toxic compared to their synthetic counterparts (Khojasteh et al., 2020).

Aescin and other phytochemicals found in *Aesculus* species exhibit multifaceted biological activities beyond their medicinal effects. These compounds display a strong antifungal (Trdá et al., 2019; Green et al., 2021) and repellent effect on the moth *Cameraria ohridella* (Kukula-Mlynarczyk et al., 2006; Ferracini et al., 2010; Oszmiański et al., 2014, 2015). Aescin triggers the innate immune response of plants by upregulating the salicylic acid pathway, thus achieving protective effects comparable to those of synthetic fungicides (Trdá et al., 2019). Additionally, *Aesculus* genotypes or species with elevated foliar concentration of aescin (Ferracini et al., 2010; Kukula-Mlynarczyk et al., 2006) or flavan-3-ols and polymeric procyanidins (Oszmiański et al., 2014, 2015) demonstrate resistance to *C. ohridella*.

Aescin, extracted industrially from *Aesculus hippocastanum* (horse chestnut) seeds, is the only phytochemical from *Aesculus* species currently known to be produced on a commercial scale. However, the seed yield varies considerably, in the range of 2–25 kg fresh weight per tree (Bellini and Nin, 2004), as a consequence of abiotic and biotic stresses. In recent decades, the leaf-mining moth *C. ohridella* has severely impacted *A. hippocastanum* populations across Europe, causing extensive leaf damage (Deschka and Dimić, 1986; Augustin et al., 2009). This pest induces premature defoliation, reducing nutrient accumulation and seed weight (Takos et al., 2008). Additionally, aescin content in seeds varies significantly due to numerous factors including genotype, environmental factors, seed maturity, and storage conditions (Profumo et al., 1987; Bellini and Nin, 2004; Kędzierski et al., 2016; Čukanović et al., 2020; Wang et al., 2023). Industrial aescin production relies on horse chestnut seeds, predominantly sourced from ornamental trees in urban environments (Bellini and Nin, 2004; Čukanović et al., 2020). Consequently, raw materials frequently contain elevated heavy metal levels, compromising pharmaceutical quality (Caldas and Machado, 2004; Čukanović et al., 2020). This underscores the need for alternative large-scale production systems. Recently, the genome of *A. chinensis* was assembled, the biosynthetic pathways for triterpenoids and coumarin glycosides were characterized and the key genes of the pathways were cloned and functionally characterized (Sun et al., 2023). However, the complexity of the metabolic pathways and the toxicity of the end products limit the possibility of their production in microbial systems (Johnston et al., 2023). Therefore, plant tissue cultures offer a promising alternative for controlled aescin production.

Somatic embryogenesis is the process by which somatic cells are reprogrammed to follow an embryogenic developmental pathway, leading to the formation of an embryo (von Arnold et al., 2002). Somatic embryos (SEs) are therefore considered the counterpart to zygotic embryos (ZEs). SEs of horse chestnut contain a considerable amount of aescin and aesculin (Profumo et al., 1991, 1994; Gastaldo et al., 1996; Ćalić et al., 2010). Therefore, embryogenic suspension cultures offer an effective platform for the sustainable, year-round production of valuable phytochemicals, including those from geographically restricted *Aesculus* species yet to be exploited in pharmaceutical applications. However, embryogenic cell suspensions have not yet been successfully established in any *Aesculus* species, and this technology remains unexploited for large-scale production of plant material for phytochemical extraction.

*A. flava* (yellow buckeye, sweet buckeye) is one such species with limited range in several U.S. states (Meyer and Hardin, 1987). Recently, a protocol for the induction of SEs from stamen filaments of *A. flava* was developed (Zdravković-Korać et al., 2019). However, SEs initiation from filament cultures exhibited highly variable and unreliable efficiency over time. The present study aimed to overcome this problem by the application of embryogenic suspension cultures. Plant cell and tissue culture provides a versatile platform for the production of plant specialized metabolites, such as taxol, ginsenosides, dyes, flavors, food ingredients, cosmetics, and other high–value phytopharmaceuticals on a commercial scale (Arya et al., 2020; Bapat et al., 2023). Embryogenic suspension cultures have proven to be a valuable tool for high biomass production, with significant potential for scalability and cost-effective automation (Välimäki et al., 2021). However, embryogenic cultures lose their embryogenic potential over time due to prolonged cultivation and particularly 2,4-dichlorophenoxyacetic acid (2,4-D) exposure, which can induce genetic or epigenetic changes in the plant cells (von Arnold et al., 2002; Garcia et al., 2019). Therefore, cryopreservation of valuable cell lines is recommended for their long-term storage and future use (Nagel et al., 2024). This safe, easy-to-perform method minimizes the risk of contamination and decline in embryogenic potential due to repeated subculturing *in vitro* and reduces culture maintenance costs and labor (Ballesteros et al., 2024). Accordingly, cryopreservation of embryogenic cell lines has been widely used in woody plant species (Ballesteros and Pence, 2019). Although various plant tissues have been successfully cryopreserved, embryogenic tissue (ET) and early-stage embryos have generally proved to be the most suitable plant material for successful recovery after storage in liquid nitrogen and subsequent thawing (Ballesteros et al., 2024). Accordingly, embryogenic calli and isolated SEs of *A. hippocastanum* have been successfully cryopreserved to date (Jekkel et al., 1998; Lambardi et al., 2005), but to our knowledge, this is the only *Aesculus* species subjected to cryopreservation. Therefore, in the present study, we aimed to develop a protocol for the successful cryopreservation of ET and SEs derived from suspension cultures of *A. flava*.

## 2 Materials and methods

### 2.1 Plant material

The inflorescences of *Aesculus flava* were collected from the tree growing in the Botanical Garden “Jevremovac”, University of Belgrade, Belgrade, Serbia. Closed flower buds (7 mm in length) were detached from the inflorescences, washed thoroughly with running tap water containing a few drops of Fairy detergent (Procter & Gamble), and surface-sterilized in 95% (v/v) ethanol for 5 min followed by 70% (v/v) ethanol for 5 min. The buds were then rinsed three times with sterile distilled water and blotted dry on sterile tissue paper. The perianth and anthers were removed and the stamen filaments (3–4 mm in length) were excised using a stereomicroscope.

### 2.2 Basal medium

The basal medium (BM) contained Murashige and Skoog (1962) macro and micro mineral salts (Lachner, Brno, Czech Republic), 2% (w/v) sucrose, 100 mg/l myo-inositol, 2 mg/l thiamine, 2 mg/l adenine, 5 mg/l nicotinic acid, 10 mg/l pantothenic acid (Sigma-Aldrich, St. Louis, MO, USA), and 200 mg/l casein hydrolysate (Torlak, Belgrade, Serbia). The pH of the media was adjusted to 5.5 with potassium hydroxide using a pH meter before sterilization, as described by Doungous et al. (2022). The media were solidified with 0.7% (w/v) agar (Torlak, Belgrade, Serbia) and sterilized in an autoclave at 114 °C (80 kPa) for 25 min.

### 2.3 Induction of friable callus and initiation of solid and liquid cultures

Filaments isolated from 2011 to 2015 were placed on solid BM containing 1 μM 2,4-D (Sigma-Aldrich) and 10 μM 6-furfurylaminopurine (Kinetin, Kin, Sigma-Aldrich) in 90-mm plastic Petri dishes (Spectar, Čačak, Serbia) and cultured in the dark for 8 weeks. This medium was designated as 1/10. The filaments were then subcultured onto solid BM medium supplemented with 400 mg/l filter-sterilized glutamine (Sigma-Aldrich) and exposed to a 16-h photoperiod with a photosynthetic flux density of 100 μmol m^−2^ s^-1^ for a further 8 weeks (Zdravković–Korać et al., 2019). The cultures were subcultured at 4-week intervals. Four to ten replications (Petri dishes) with 15 filaments each were prepared per year. Filaments producing ET were recorded at the end of a 16-week period. The frequency of SEs regeneration was calculated per Petri dish.

Friable callus induction protocols were adjusted over time, incorporating liquid media for experiments from 2019 to 2024, as detailed below. The effect of medium consistency on ET initiation was compared between liquid and solid cultures. For this experiment, freshly isolated filaments were cultured in the dark on solid 1/10 medium for 4 weeks. Subsequently, whole explants, which showed no signs of necrosis, were used to initiate both liquid and solid cultures. For both types of cultures, 500 mg of the explants were inoculated into 25 ml of liquid or solid 1/10 medium. The liquid cultures were cultivated in 100-ml Erlenmeyer flasks and shaken at 95 rpm, while the solid cultures were cultivated in Petri dishes. After 6 weeks, the explants from the solid cultures were subcultured onto fresh solid 1/10 medium, while the liquid cultures were refreshed with an additional 25 ml of liquid 1/10 medium. Both culture types were maintained in the dark throughout the 16-week period. Five replicates, each with ten subsamples (Erlenmeyer flasks/Petri dishes), were prepared annually for each culture type. The embryogenic cultures were recorded at the end of the 16-week period. The frequency of SEs regeneration, calculated as the percentage of explants forming SEs, was determined per replicate.

In this study, the following terms are used: ET – any proliferating tissue that forms cotyledonary somatic embryos after transfer to proliferation medium; embryogenic cells aggregates (ECAs) – the characteristic morphological units observed macroscopically and under the stereomicroscope as white to pale-yellow, nodular or irregularly shaped proliferating structures; proembryogenic masses (PEMs) – histologically confirmed nodular structures composed of small, isodiametric, densely cytoplasmic cells, corresponding to the earliest organized stage of the embryogenic pathway, which subsequently generated somatic embryos and/or other embryogenic nodules. The terms ‘ECAs’ and ‘PEMs’ are used interchangeably when histological evidence is provided; otherwise, the broader and more descriptive term ‘ECAs’ is preferred.

### 2.4 Histological analysis

To understand the transition of friable callus to ET and to determine the nature of ECAs, explants containing friable callus, embryogenic callus, and the ECAs were sampled for histological analysis immediately after their appearance in the liquid cultures. Isolated material was fixed in FAA (formaldehyde: acetic acid: 70% ethanol 2:1:17) at 4°C for 48 h. Following dehydration in graded ethanol series, the samples were cleared in xylene, embedded in Histowax^®^ (Histolab Products AB, Gothenburg, Sweden) at 58°C and sectioned at 5–7 μm. Sections were stained with 0.05% toluidine blue O in 0.1 M phosphate buffer at pH 6.8 (O’Brien et al., 1964), mounted in Canada balsam and observed under a Zeiss Axiovert light microscope (Carl Zeiss GmbH, Göttingen, Germany).

### 2.5 Establishment of suspension cultures

At the end of a 16-week period, the liquid cultures were sequentially sieved through 0.9 mm and 0.05 mm nylon meshes (Macrokun, Shijiazhuang, China) to remove cell debris and necrotic remnants of the filaments. The resulting ECAs (0.05–0.9 mm) were collected and used to initiate fine suspensions by inoculating 100 mg ECAs per 100 ml of liquid 1/10 medium into 250-ml Erlenmeyer flasks. The suspension cultures were shaken in the dark at 95 rpm.

To monitor growth and optimize subcultivation intervals, the proliferation of 0.6–0.9 mm ECAs in liquid 1/10 medium was investigated. Fifty mg of ECAs were inoculated into 50 ml of liquid 1/10 medium and cultured as described above. Each week, three suspensions were randomly selected and filtered through a 0.05 mm filter, blotted dry on tissue paper for 10 min and the fresh weight (FW) of ECAs/SEs was determined. The material was then dried at 60°C to constant weight and the dry weight (DW) was determined. The experiment lasted nine weeks and was repeated three times, comprising three suspensions per replicate (n = 9).

ECAs proliferation and SEs regeneration from ECAs were determined in three lines (9, 12, and 68) selected on the basis of their low, moderate and high embryogenic capacity. Approximately 50 mg of ECAs (0.6–0.9 mm) was cultured in liquid 1/10 medium, and FW and SEs number were recorded after 4 weeks. The relative FW increase was calculated using the formula (FW_4_–FW_0_)/FW_0_, where FW_0_ is the initial FW of the inoculum and FW_4_ is the FW after four weeks of culture. The SEs number was normalized to 50 mg initial inoculum. For each cell line, three replicates with three suspensions each were prepared (n = 9 per line, total n = 27).

The suspensions were maintained by regular filtration through a 0.6 mm filter at two-week intervals to retain ECAs ≤ 0.6 mm. The filtrates were diluted with the same volume of liquid 1/10 medium. ECAs obtained from different regeneration events, i.e., in different Erlenmeyer flasks, were maintained as different cell lines.

### 2.6 Suspension fractionation, proliferation and embryogenic capacity of size fractions

To analyze the ECAs size profile, ECA suspensions were simultaneously filtered through a set of nylon filters (pore sizes 0.05, 0.3, 0.6, and 0.9 mm; Macrokun, Shijiazhuang, China) and a 2.38 mm stainless steel filter (Sigma-Aldrich), then washed thoroughly with liquid 1/10 medium to obtain ECA fractions 0.05–0.3 mm, 0.3–0.6 mm, 0.6–0.9 mm, 0.9–2.38 mm and > 2.38 mm. Each fraction was drained for 30 minutes on a 0.05 mm nylon filter placed over a thick layer of sterile tissue paper in separate, sterile, closed glass Petri dishes. The FW of each fraction was measured and its proportion per suspension was calculated.

To evaluate the proliferative and embryogenic potential of each ECA fraction, approximately 50 mg of ECAs from each fraction was cultured on a pre-wetted, pre-weighed 0.05 mm nylon filter placed over solid BM medium with 0.05 μM 2,4-D and 5 μM Kin in plastic Petri dishes and incubated in the dark for four weeks. This medium, designated as 0.05/5, was successfully used for SEs proliferation in previous studies (Radojević et al., 1989; Ćalić et al., 2005; Zdravković-Korać et al., 2008, 2019). The FW was measured after four weeks, and the FW increase was calculated as described in subsection 2.5. The number of SEs was determined at the start and after four weeks, then normalized to 50 mg of initial inoculum. SEs were classified according to their stage of development: globular/heart-shaped (GSE/HSE), torpedo-shaped (TSE), late TSE (LTSE), and cotyledonary (CSE). Malformed SEs were also counted. Five replicates with three samples (Petri dishes) per size fraction were prepared (n = 15).

### 2.7 Impact of sucrose on proliferation and dry matter increase of CSEs

CSEs (1 cm in length) obtained from previous experiments were cultured for four weeks in the dark on solid 0.05/5 medium supplemented with 2, 4, 6, 8 or 10% sucrose. Ten CSEs were cultured per Petri dish, with all CSEs from one Petri dish measured as a single sample. Their FW was determined at the start and end of the 4-week cultivation period. After FW measurement, CSEs were frozen in liquid nitrogen and stored in a freezer at –80°C. DW was measured after freeze-drying. The FW increase was calculated as described in subsection 2.5. Dry matter percentage was calculated using the formula: (DW/FW) x 100. In order to simultaneously estimate the FW increase and the percentage of dry matter, an index fresh-to-dry weight (FDW) was calculated as: FW increase × dry matter percentage. Five replicates with 3 samples (Petri dishes) per treatment were used (n = 15).

### 2.8 Cryopreservation of ECAs/SEs

In order to preserve the embryogenic capacity of ECAs/SEs, they were cryopreserved following the modified plant cell line cryopreservation procedure described in Schumacher et al. (2015). ECAs/SEs (0.05–0.9 mm) harvested from the suspensions in the exponential growth phase and filtered through a nylon filter were used as the starting material for cryopreservation. The cryopreservation protocol, combining encapsulation and one-step freezing, required a five-day preparation period. On the first day, alginate beads were prepared according to the following procedure: cell suspension pellet (4 ml) was transferred into a sterile Falcon tube (15 ml) using a spatula and 8 ml of 3% Na-alginate was added. After capping the Falcon tube, the cells were immersed in the alginate by careful mixing. Using sterile pipettes with tips shortened by ~10 mm, beads were prepared by dispensing Na-alginate solution containing suspended cells, into 100 ml of liquid BM medium containing 100 mM calcium chloride in a 250-ml glass jar. The alginate drops were polymerized for 20 min in a calcium-enriched liquid medium until the beads were fully formed. After 20 min of polymerization, the calcium-containing liquid BM medium was decanted and the beads were washed twice with the fresh BM medium. One hundred beads were transferred to 100 ml of liquid BM medium in a 300-ml Erlenmeyer flask and shaken for three days under standard growth conditions. After three days, the culture medium was decanted, and the beads were transferred in 100 ml of liquid BM medium with 0.9 M sorbitol, then incubated for two days at 4°C. On the fifth day, 5 ml of undiluted DMSO was added to the Erlenmeyer flask containing alginate beads in 100 ml BM medium with 0.9 M sorbitol (5% v/v), and the beads were incubated at 4°C for a further 60 min. After draining the medium, the beads were decanted in a sterile Petri dish and five beads were transferred to a 2-ml cryovial using sterile forceps. Cryovials were then placed in a MrFrosty^®^ Nalgene freezing container (Sigma-Aldrich) with isopropanol (cooling rate –1°C/min, capacity 18 vials) and kept at –80°C for one hour (LN-). After that, the cryovials were directly transferred to liquid nitrogen (–196°C, LN+). The samples were stored in liquid nitrogen for at least one day. Non-cryopreserved encapsulated alginate beads served as the control. Both LN- and LN+ samples were thawed in a water bath (40°C) for 3 min. Control and thawed beads were then carefully immersed in the solid 0.05/5 medium in Petri dishes (Ø 9 cm, 25 ml per dish). After 24 h, the beads were transferred to fresh solid 0.05/5 medium, and ECAs regrowth and SEs regeneration from the beads were recorded after 8 weeks. Regrowth from the beads is measured as percent of beads enveloped with new regenerated ECAs/SEs.

### 2.9 LC/MS compound identification

Metabolite profiling was conducted on ECAs/SEs at successive developmental stages: ECA, GSE, HSE, TSE, and LTSE, obtained from cultures grown for four weeks on 0.05/5 medium with 2% sucrose, and CSEs cultivated on solid 0.05/5 medium with 2% or 8% sucrose (designated as CSA–2 and CSA–8, respectively). ZEs of *A. flava* were used for comparison. The ripe fruits of *A. flava* were collected 22 weeks after anthesis from the same mother tree as the inflorescences. The pericarps and endocarps of the fruits were removed, and the remaining seeds (cotyledons + embryo axes) were used for analysis. Three biological replicates, each consisting of five pooled ZEs, were prepared.

The plant material was frozen in liquid nitrogen and stored at –80°C until use. It was then freeze–dried and pulverized to a very fine powder in liquid nitrogen. The extraction procedure was performed according to Dias et al. (2022), with some minor modifications. One g (DW) of each sample was extracted with 50 ml of 96% ethanol, and sonicated for 20 min in an ultrasonic bath (Sonorex Digiplus, Bandelin, Berlin, Germany) at a peak ultrasonic power of 192 W (nominal power of 48 W). The samples were then centrifuged at 3,000 rpm for 15 min, the supernatants were recovered using a pipette, and evaporated to dryness in a concentrator (Concentrator 5301, Eppendorf, Germany). The dried ethanolic extracts were weighed and dissolved in HPLC–grade absolute methanol (J.T. Baker) to a concentration of 10 mg/ml. The aescin standard (Fluka, Buch, Switzerland) was prepared in absolute methanol at 1 mg/ml. The aliquots were filtered through a syringe filter (0.22 µm) prior to LC/MS analysis. Three biological replicates per ECA/SE developmental stage were prepared.

The LC/MS analysis of the extracts was conducted using Thermo Scientific™ Vanquish™ Core HPLC system coupled to the Orbitrap Exploris 120 mass spectrometer (San Jose, CA, USA). All LC/MS parameters are explained in detail in Stojković et al. (2024). The obtained MS data were processed and analyzed using R Studio software (version 2023.09.1, build 494) using enviPick and xcms R packages (Zengin et al., 2020). Identification of bioactive compounds was achieved based on their chromatographic behavior and HRMS/MS^2^ data, with comparisons made to standard compounds, when available, and literature data about metabolites from *Aesculus* species. The literature review was performed by searching the SciFinder database (CAS SciFinder chemical compound database, 2025) using suggested molecular formulas and keywords. Data acquisition was carried out using Xcalibur^®^ data system (Thermo Finnigan, San Jose, CA, USA).

### 2.10 Recordings and statistical analysis

A completely randomized design was used for the placement of the cultures. Percentage data were subjected to angular transformation and SEs number data to square root transformation prior to analysis, followed by inverse transformation for presentation. Data were subjected to a standard analysis of variance, and means were separated using the LSD *post-hoc* test at *P* ≤ 0.05.

To differentiate between samples in the LC/MS analysis, hierarchical cluster analysis (HCA) plots were constructed in Morpheus software (Broad Institute, 2025), based on the Spearman method of cluster agglomeration, adopting the average linkage method. Variables were relative peak areas (relative abundance) obtained from full-scan MS.

## 3 Results

### 3.1 The efficiency of embryogenic tissue induction: solid vs. liquid cultures

Friable callus in the present study was initiated by culturing *A. flava* stamen filaments on solid medium for four weeks, in order to localize contamination and prevent significant loss of the material. The effect of the consistency of the medium was then tested using 4-week-old, non–necrotic calli. ET was not observed during this initial 4-week cultivation.

**Figure 1:**
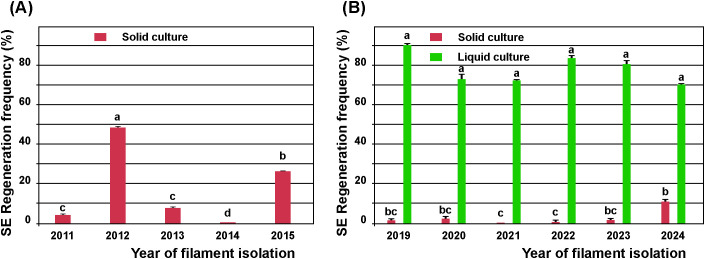
Variation in the frequency of regeneration of somatic embryos (SEs) from the stamen filaments of *Aesculus flava* over eleven years: effect of the consistency of the medium. **(A)** Filaments isolated in 2011–2015 were cultured for eight weeks in darkness on solid medium with 1 μM 2,4-D + 10 μM Kin (1/10) and then subcultured on solid medium without plant growth regulators and exposed to a 16–h photoperiod for a further eight weeks. Four to ten replicates with 15 filaments were prepared per year. SEs regeneration frequency was calculated per Petri dish. **(B)** Filaments isolated in 2019–2024 were cultured in the dark on solid 1/10 medium for four weeks and then subcultured to either solid or liquid 1/10 medium, using 500 mg of whole callusing filaments per culture for a further 12 weeks. Five replicates of ten samples each (Erlenmeyer flask/Petri dish) were prepared annually for each culture type. The presence of embryogenic tissue was recorded 16 weeks after culture initiation. The SEs regeneration frequency was calculated per replicate. Data represent the mean ± standard error. Treatments labelled with the same letter do not differ significantly (p ≤ 0.05) according to the LSD test.

The frequency of ET induction from the friable calli derived from *A. flava* stamen filaments cultured on solid 1/10 medium varied widely over five years (2011–2015), ranging from 0% in 2014 to a peak of 48.68 ± 0.07% in 2012 (**Figure 1A**). To prevent necrosis of friable calli and increase nutrient availability and ET induction rates, whole, non-necrotic callus-forming explants were cultured in liquid 1/10 medium and compared to those cultured on solid 1/10 medium. In the period 2019–2024, the frequency of ET initiation from the same amount of callusing filaments was significantly higher (p ≤ 0.00001) in liquid than on solid medium (**Figure 1B**). The frequency of ET induction in liquid medium varied from 70.4 ± 0.24% to 90.45 ± 2.28%, while in solid cultures it ranged from 0% to 10.95 ± 0.24% (**Figure 1B**). Therefore, ET initiation in liquid medium was more efficient and reliable than on solid medium of the same composition. ET induction frequencies for solid cultures in 2011–2015 were higher than those in 2019–2024 because a greater quantity of material was cultured per Petri dish (approximately 2 g vs. 0.5 g).

**Figure 2:**
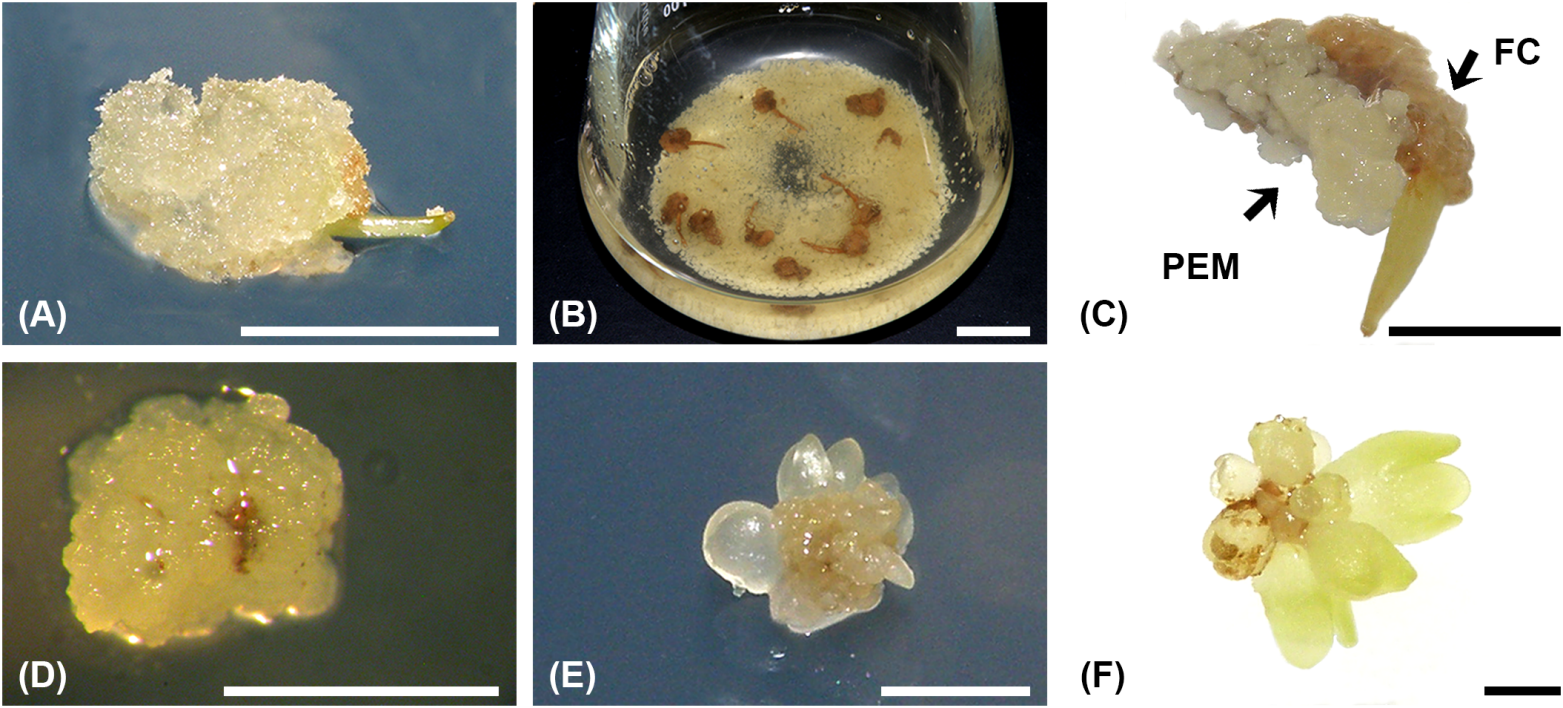
Formation of embryogenic cell aggregates (ECAs) and SEs regeneration in the liquid culture of *A. flava* friable callus. **(A)** Friable callus formed from the filament cultured in the dark on solid 1/10 medium for four weeks. **(B)** ECAs nine weeks after liquid culture initiation. **(C)** Formation of proembryogenic masses (PEMs) from friable callus (FC) cultured on solid 1/10 medium for eight weeks. **(D)** An ECA obtained after sieving the liquid culture. **(E, F)** SEs regeneration from the same ECA after two weeks **(E)** and three weeks **(F)** of culture on solid MS medium supplemented with 0.05 μM 2,4-D + 5 μM Kin. ECAs – the characteristic morphological units observed macroscopically and under the stereomicroscope as white to pale-yellow, nodular or irregularly shaped proliferating structures; PEMs – histologically confirmed nodular structures composed of small, isodiametric, densely cytoplasmic cells, corresponding to the earliest organized stage of the embryogenic pathway, which subsequently generated SEs and/or other embryogenic nodules. Scale bars: a–c = 10 mm, d–f = 1 mm.

In liquid medium, cells detached from the surface of friable calli (**Figure 2A**) and dispersed in the medium, while the enlarged filament body remained compact (**Figure 2B**). However, only a slight proliferation of dispersed cells was observed, followed by necrosis of the filament body within 1–2 weeks. Maceration of the explants, to enhance nutrient availability to the cells, did not prevent explant necrosis or improve cell proliferation and culture density (data not shown). ET appeared 4–8 weeks after the initiation of liquid culture and proliferated rapidly and vigorously (**Figure 2B**). Concomitantly, the same type of ET was observed on filaments cultured on solid 1/10 medium (**Figure 2C**). The ET was globular and segmented, with a brown necrotic zone in the center (**Figure 2D**). As appeared, ET immediately started to regenerate SEs in both solid and liquid cultures (**Figures 2E, F**).

### 3.2 Histological analysis of friable callus and ECAs

**Figure 3:**
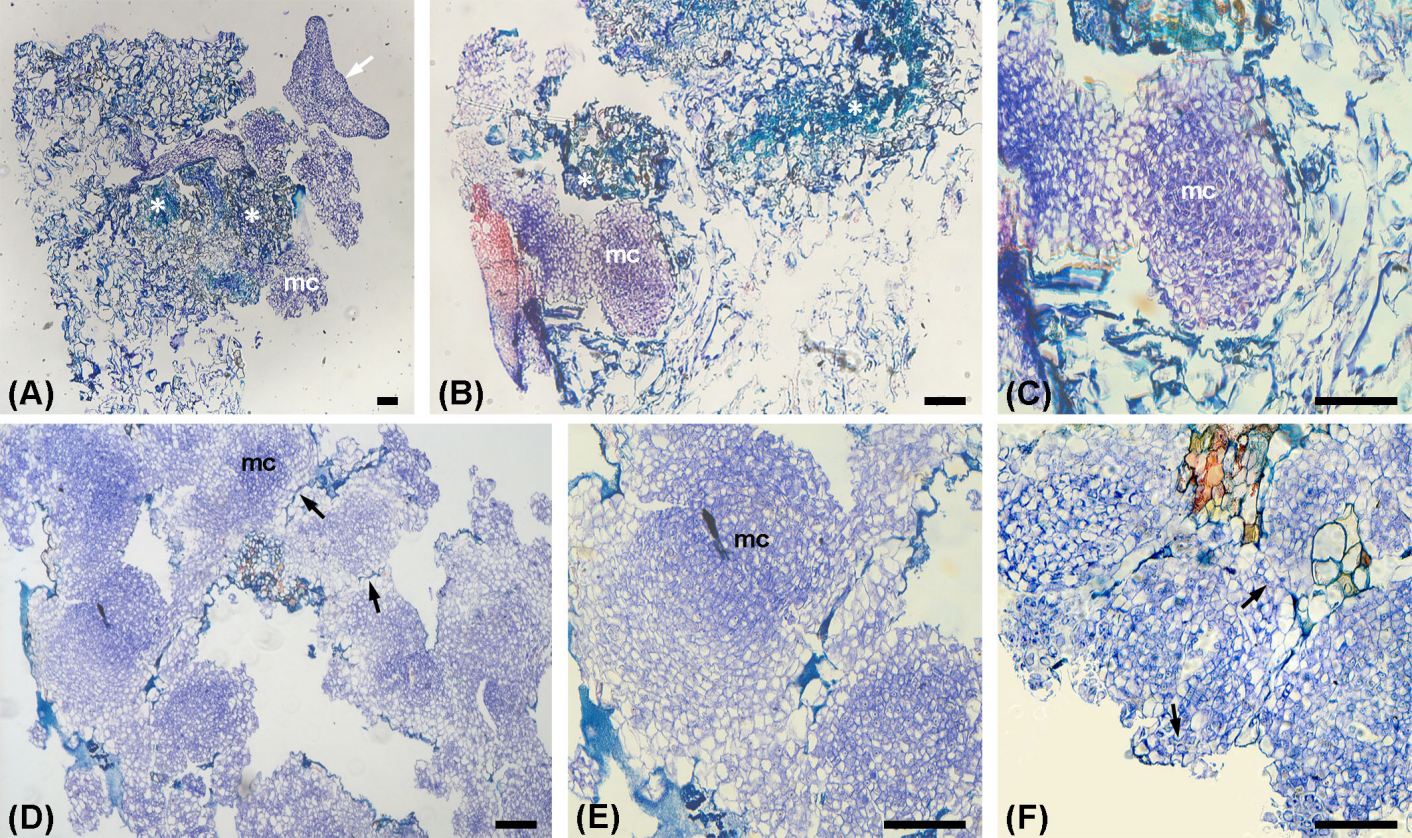
Histological analysis of friable-to-embryogenic callus transition and ECAs formation. **(A)** Section of friable embryogenic callus cultured in 1/10 medium, with pores and necrotic regions (*asterisks*), showing isolated clusters of meristematic cells (*mc*) and embryogenic structures with distinct protoderm (*arrow*). **(B, C)** Embryogenic callus with clusters of tightly packed meristematic cells (*mc*) surrounded by irregularly shaped vacuolated cells; **(C)** - detail of **(B) (D, E)** ECAs cultured on solid 0.05/5 medium for one week; **(E)** – detail of **(D)** Note segmentation lines (*arrows*) between adjacent nodules containing meristematic clumps (*mc*). **(F)** Proliferation of ECAa after 2 weeks of culture. Note irregularly shaped clusters of meristematic cells showing signs of tissue disorganization at their periphery (*arrows*). ECAs – the characteristic morphological units observed macroscopically and under the stereomicroscope as white to pale-yellow, nodular or irregularly shaped proliferating structures. Scale bars = 100 μm.

Histological analysis showed that a brownish friable callus mostly contained fragments of necrotic tissue in which isolated clusters of highly meristematic cells and SEs structures with distinct protoderm were observed (**Figure 3A**). Regions with thick-walled cells exhibiting meristematic characteristics and intense mitotic activity were surrounded by thin-walled, highly vacuolated and irregularly shaped parenchymatous cells with large intercellular spaces (**Figures 3B, C**).

Histological analysis of ECAs revealed the presence of friable nodules, composed of two histological regions with a gradual transition between the two: more internally positioned meristematic cells, organized in PEMs and larger more vacuolated parenchymatous cells at the periphery (**Figure 3D**). Differently sized intercellular spaces were present at low frequency. PEMs consisted of small isodiametric cells with relatively dense cytoplasm, small vacuoles, large nuclei with prominent nucleoli, and a high nucleus–to–cell–area ratio (**Figure 3E**). A distinct protoderm was not observed.

Proliferating ECAs comprised meristematic cell clusters, composed of clumps of densely packed, cytoplasm-rich cells (**Figure 3F**). Meristematic cell clusters varied in size and shape and were also characterized by the presence of two histological regions with a gradual transition between them. More irregularly shaped clusters often exhibited signs of tissue disorganization at their periphery and cell degradation of the outermost vacuolated cells (**Figures 3E, F**). Sections through ECAs revealed their partial segmentation, with adjacent meristematic cell clusters clearly delineated from one another (**Figures 3E, F**).

### 3.3 Initiation and maintenance of the suspension cultures

**Figure 4:**
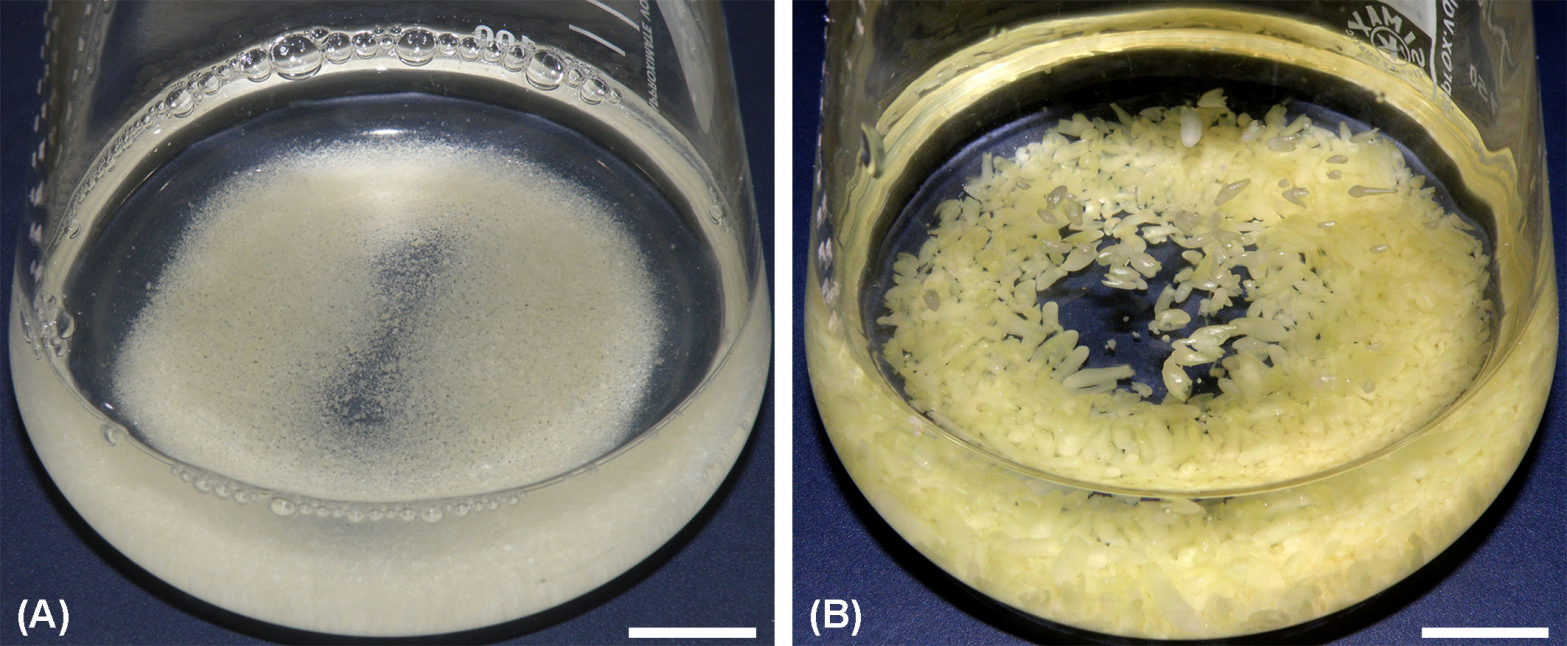
Initiation of a suspension culture. **(A)** ECAs 0.6–0.9 mm immediately after filtration. **(B)** The same culture after two weeks of cultivation in the dark at 95 rpm. ECAs – the characteristic morphological units observed macroscopically and under the stereomicroscope as white to pale-yellow, nodular or irregularly shaped proliferating structures. Scale bars = 10 mm.

After filtration, liquid cultures were cleared of the remains of necrotic filaments and lysed cells and obtain fine ECA suspensions (**Figure 4A**) that exhibited sustained proliferation and efficient SEs regeneration (**Figure 4B**). In suspension cultures, ECAs not only enlarged but also continuously released smaller cell aggregates, resulting in a wide range of ECA diameters. In addition, newly formed SEs proliferated via secondary somatic embryogenesis, thus producing cultures that contained SEs from globular to advanced stages of development (**Figure 4B**). Filtration was essential for the efficient maintenance of suspension cultures, as ECAs larger than 2.38 mm became necrotic easily and produced fused or malformed SEs. Also, TSEs and CSEs became hyperhydrated when grown in liquid medium for an extended period of time.

**Figure 5:**
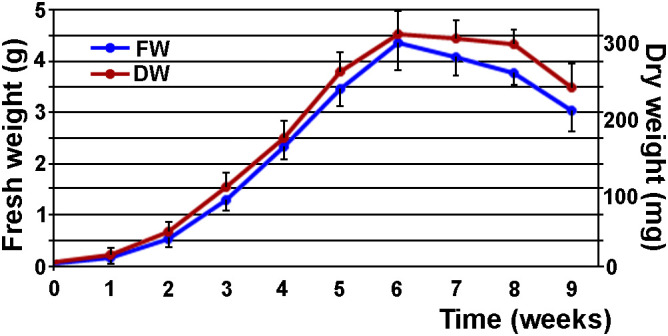
Proliferation of 0.6–0.9 mm ECAs in liquid medium over nine weeks of cultivation, assessed using: fresh weight (FW), and dry weight (DW) as indicators. Fifty mg of ECAs were inoculated into 50 ml of liquid medium 1/10 and shaken in the dark at 95 rpm. Each week, three suspensions were sieved through a 0.05 mm mash, blotted dry and the FW measured. The samples were dried to a constant weight and the DW was determined. The experiment was repeated three times (n = 9). Data represent the mean ± standard deviation.

The growth curve showed that the suspensions were in exponential growth 2 weeks after the initiation of the suspensions (**Figure 5**). The cultures exhibited significant proliferation, reaching maximum FW and DW of 4.336 ± 0.417 g and 0.247 ± 0.020 g, respectively, 6 weeks after the initiation of the suspensions (**Figure 5**). Therefore, suspensions were maintained by filtration through a 0.6 mm filter at two-week intervals, and the filtrate was refreshed with an equal volume of 1/10 medium. Occasionally, new suspensions were initiated following the same procedure as the initial setup.

### 3.4 Proliferation and SEs regeneration from ECAs of different cell lines

**Figure 6:**
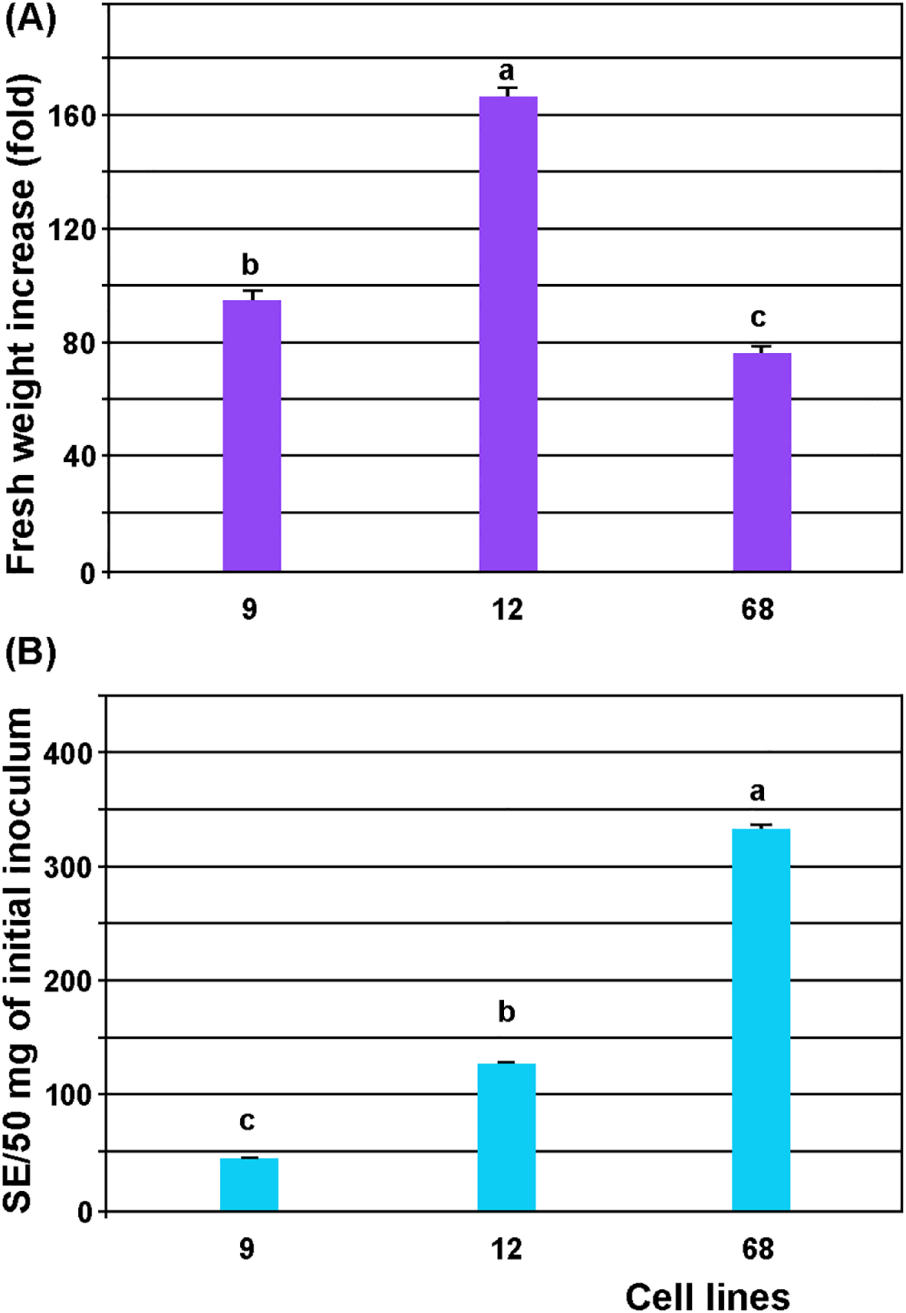
Proliferation and SEs regeneration from ECAs of the three selected lines (9, 12 and 68). **(A)** Increase in fresh weight, **(B)** number of somatic embryos (SEs) per 50 mg of initial inoculum. Approximately 50 mg ECAs (0.6–0.9 mm) were cultured in 50 ml of liquid medium 1/10 and shaken in the dark at 95 rpm. The FW and number of SEs were recorded after four weeks. The number of SEs was normalized to 50 mg initial inoculum weight. For each cell line, three replicates with three suspensions each (n = 9) were prepared. Data represent the mean ± standard error. Treatments labelled with the same letter do not differ significantly according to the LSD test (p≤ 0.05).

Numerous cell lines were established from independent regeneration events. Although they originated from the same genotype (tree), their proliferative and embryogenic capacities differed considerably. Significant statistical differences were observed in FW increase (p ≤ 0.000001) and mean SEs number (p ≤ 0.01) among the three selected lines (9, 12 and 68) after four weeks of cultivation in liquid 1/10 MS medium. The initial FW of ECAs increased 76.3–166.8-fold (**Figure 6A**), while 45.19–333.77 SEs were obtained per initial inoculum of 50 mg in these lines (**Figure 6B**). Since the suspensions of line 68 exhibited the highest embryogenic capacity in addition to a high proliferation rate, this line was selected for further optimization of proliferation and SEs regeneration by filtration.

ECAs of some lines showed a low proliferative/regenerative capacity, as the ECAs were rapidly depleted by SEs regeneration, while the regenerated SEs progressed into advanced developmental stages and lost their capacity for secondary somatic embryogenesis. Nevertheless, most lines maintained a high but variable proliferative and SEs regeneration capacity for 6–9 months after ET initiation. Highly proliferative, embryogenic suspensions had an intense yellow appearance (**Figure 4B**), while the color of suspensions that had lost their embryogenic capacity became light cream.

### 3.5 Size-fractionation of suspensions

**Figure 7:**
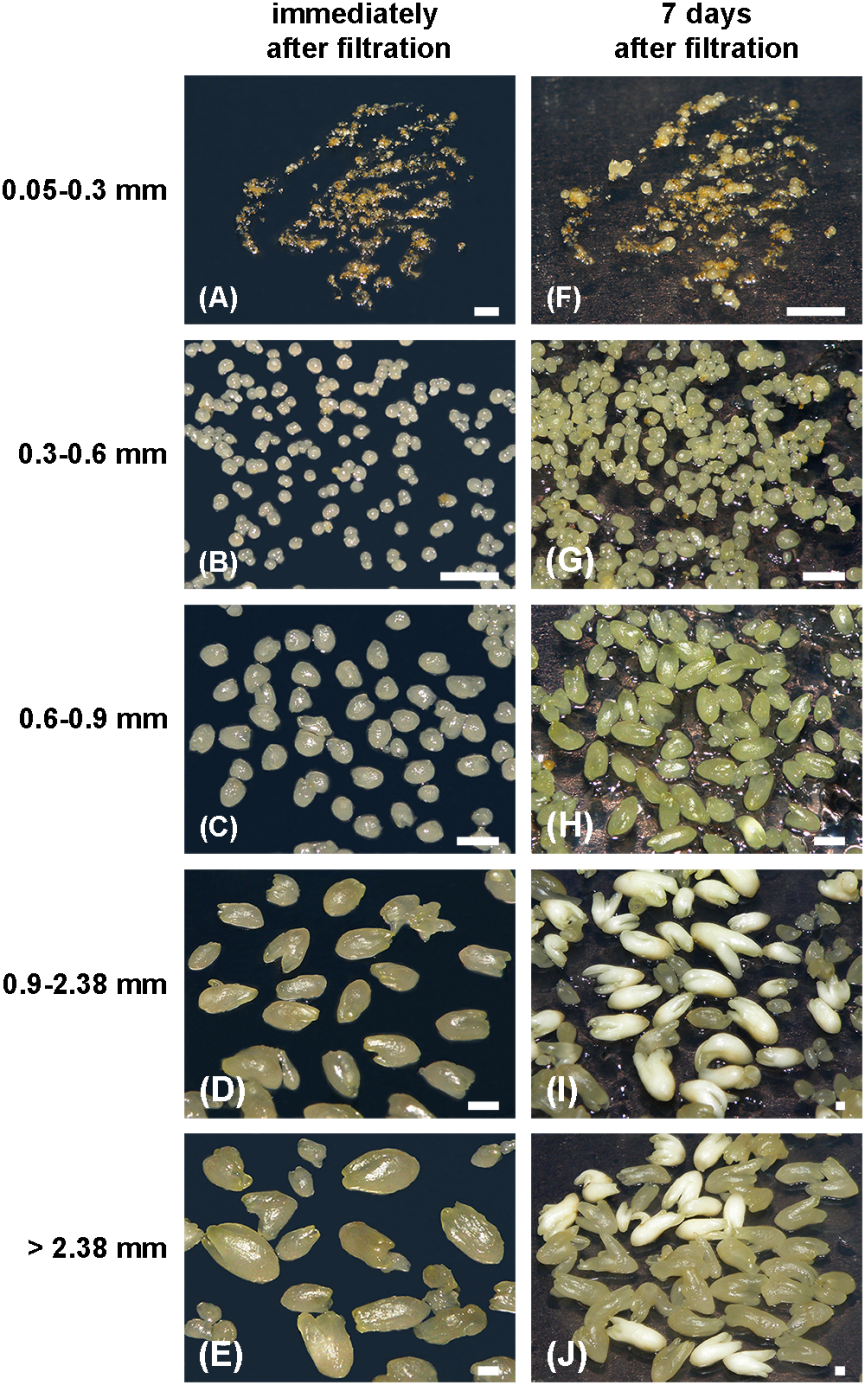
Size fractions of a suspension sieved through a set of filters of 0.05, 0.3, 0.6, 0.9 and 2.38 mm. **(A, F)** 0.05–0.3 mm fraction with ECAs, **(B, G)** 0.3–0.6 fraction with GSEs, **(C, H)** 0.6–0.9 fraction with HSEs, **(D-I)** 0.9–2.38 fraction with TSEs, and **(E, J)** SEs > 2.38 mm, immediately after sieving **(A-E)** and after one week of cultivation on solid 0.05/5 medium **(F-J)**. Scale bars: a–j = 1 mm.

Filtration of suspensions enhanced SEs release from ECAs and prevented the formation of large aggregates, so that the suspensions contained predominantly free SEs and small ECAs (**Figure 4**). Furthermore, filtration enabled SEs separation by developmental stage (**Figures 7A–E**). Immediately after filtration, the 0.05–0.3 mm fraction primarily contained irregularly shaped ECAs and a few preglobular and globular structures, visible with the aid of a stereomicroscope (**Figure 7A**). The 0.3–0.6 mm, 0.6–0.9 mm and 0.9–2.38 mm fractions contained predominantly GSEs (**Figure 7B**), HSEs (**Figure 7C**) and early TSEs (**Figure 7D**), respectively. The > 2.38 mm fraction consisted mainly of LSEs and CSEs (**Figure 7E**), though some of these SEs were malformed. SEs in each fraction continued to develop synchronously on solid 0.05/5 medium (**Figures 7F–J**).

All ECA/SE fractions tested in the present study exhibited high proliferation rates after four weeks of cultivation on solid 0.05/5 medium. The initial size of ECAs/SEs significantly influenced their proliferation, i.e., biomass production (p ≤ 0.01). The 0.3–0.6 mm fraction showed the highest FW increase (116.49-fold), followed by the 0.05–0.3 mm and 0.6–0.9 mm fractions (89.85- and 85.40-fold, respectively), whereas larger ECAs/SEs had a significantly lower increase in FW, up to 36.47-fold (**Table 1**).

**Table 1:** Proliferation and somatic embryos (SEs) regeneration capacity of embryogenic cell aggregates (ECAs) of different fractions.

ECAs/SEs (mm)	SEs stage	SEs no. initial	FW fold increase	SEs no. after 4 weeks
0.05–0.3	ECA	1.54 ± 0.07 e	89.85 ± 2.54 b	975.95 ± 0.31 a
0.3–0.6	GSE	106.60 ± 0.04 a	116.49 ± 2.85 a	746.03 ± 0.35 b
0.6–0.9	HSE	71.93 ± 0.01 b	85.40 ± 1.68 b	329.10 ± 0.16 c
0.9–2.38	TSE	26.68 ± 0.07 c	36.47 ± 3.21 c	56.78 ± 0.05 d
>2.38	LTSE	9.79 ± 0.01 d	14.35 ± 1.58 d	18.85 ± 0.03 e
ECAs/SEs size		p ≤ 0.01	p ≤ 0.01	p ≤ 0.01

ECAs/SEs of different fractions (~50 mg) were plated on a 0.05-mm mesh placed over solid 0.05/5 medium and cultured in the dark for four weeks. The number of SEs was determined at the start and after four weeks of culture and normalized to 50 mg initial inoculum. Fresh weight (FW) was measured after four weeks of culture and the relative FW increase was calculated using the formula (FW_4_–FW_0_)/FW_0_. Five replicates, each with 3 samples per fraction, were prepared (n = 15). Data represent the mean values ± standard error. Means labelled with the same letter do not differ significantly (p ≤ 0.05) according to Fisher’s LSD test. GSE, globular SE; HSE, heart-shaped SE; TSE, torpedo SE; LTSE, late torpedo SE; CSE, cotyledonary SE.

Furthermore, a high embryogenic response was observed, and mean SEs number was significantly affected by ECA size (p ≤ 0.01). Although PEMs or GSEs were rarely observed in the 0.05–0.3 mm fraction immediately after sieving, this fraction yielded the highest number of SEs (975.95 ± 0.31 per 50 mg inoculum) after four weeks of cultivation on solid 0.05/5 medium (**Table 1**). The mean number of SEs decreased with increasing ECAs/SEs size, reaching only 18.85 ± 0.03 for ECAs/SEs > 2.38 mm (**Table 1**).

The 0.05–0.3 mm ECA/SE fraction regenerated mainly GSEs + HSEs (57.10%) and less than 2% of LTSEs + CSEs for four weeks (**Table 2**). As expected, the percentage of GSEs and HSEs decreased, while that of TSEs and CSEs increased with increasing initial ECAs/SEs size. However, the percentage of malformed SEs also increased significantly with increasing ECAs/SEs size and was over 25% for ECAs/SEs > 2.38 mm (**Table 2**). Thus, ECAs/SEs < 2.38 mm exhibited a high proliferation rate, high embryogenic capacity, the ability to regenerate healthy LTSEs and CSEs, and an acceptable SEs malformation rate of 2.7–16% (**Table 2**). The CSEs obtained from this experiment were subcultured on solid 0.05/5 medium with 2–10% sucrose.

**Table 2:** Proportions of SEs in successive stages of development, expressed as a percentage of the total number of SEs.

ECAs (mm)	GSEs + HSEs %	TSEs %	LTSEs %	CSEs %	Malformed SEs %
0.05–0.3	57.10 ± 0.04 a	39.97 ± 0.04 b	1.54 ± 0.01 e	0.32 ± 0.02 e	2.68 ± 0.03 d
0.3–0.6	48.15 ± 0.03 b	46.78 ± 0.03 a	4.11 ± 0.01 d	0.96 ± 0.03 d	3.59 ± 0.02 d
0.6–0.9	36.87 ± 0.03 c	47.42 ± 0.03 a	10.00 ± 0.01 c	5.13 ± 0.03 c	8.34 ± 0.02 c
0.9–2.38	30.20 ± 0.09 d	36.77 ± 0.06 b	18.31 ± 0.01 b	13.09 ± 0.01 b	15.96 ± 0.02 b
>2.38	15.25 ± 0.13 e	23.69 ± 0.07 c	25.99 ± 0.07 a	32.41 ± 0.05 a	25.22 ± 0.04 a
ECAs/SE size	p ≤ 0.01	p ≤ 0.000001	p ≤ 0.01	p ≤ 0.01	p ≤ 0.01

Five replicates with 3 samples per treatment were used (n = 15). Data represent the mean values ± standard error. Means labelled with the same letter are not significantly different (p ≤ 0.05) according to Fisher’s LSD test. GSE, globular SEs; HSE, heart-shaped SEs;TSE, torpedo SEs; LTSE, late torpedo SEs; CSE, cotyledonary SEs. Refers to the data in **Table 1**

### 3.6 Effect of sucrose concentration on FW and dry matter content of CSEs

FW of CSEs increased by 7–11.5-fold on a 0.05/5 medium supplemented with 2–10% sucrose for four weeks (**Table 3**). FW increased with sucrose concentration, with the highest value observed for CSEs cultured on medium supplemented with 6% sucrose, although no significant differences in FW were found among treatments (**Table 3**). Dry matter also increased significantly (p ≤ 0.01) with sucrose concentration, with the highest values of 20.70% and 22.11% for CSEs cultured on medium supplemented with 8% and 10% sucrose, respectively (**Table 3**). The index FDW, which was introduced to account for both biomass production and dry matter content, was also significantly affected by sucrose concentration (p ≤ 0.0001), with the highest value for CSEs cultured on medium with 8% sucrose (**Table 3**); thus, these CSEs were used for chemical analysis, along with those cultured on medium with 2% sucrose for comparison. During 4-week cultivation of CSEs on 0.05/5 medium, secondary SEs were only seldom observed.

**Table 3:** Effect of sucrose on FW, dry matter percentage (DW/FW) and an index fresh-to-dry weight (FDW) of CSEs cultured for four weeks on 0.05/5 medium with 2–10% sucrose.

Sucrose %	CSEs FW fold increase	DW/FW %	Index FDW
2	7.08 ± 0.66	9.83 ± 0.002 d	221.96 ± 18.21 c
4	9.93 ± 1.09	12.93 ± 0.003 c	358.03 ± 32.47 b
6	11.54 ± 1.22	16.03 ± 0.009 b	463.00 ± 38.21 ab
8	10.80 ± 1.24	20.70 ± 0.005 a	501.45 ± 50.78 a
10	9.29 ± 1.12	22.11 ± 0.015 a	439.17 ± 46.87 ab
Sucrose concentration	NS	p ≤ 0.01	p ≤ 0.0001

Dry matter percentage was calculated using the formula: (DW/FW) x 100, while the index FDW was calculated as: FW increase × dry matter percentage. The data represent the mean values ± standard error. Five replicates with 3 samples (Petri dishes) per treatment were prepared (n = 15). Means labelled with the same letter are not significantly different (p ≤ 0.05) according to Fisher’s LSD test. NS, not significant.

### 3.7 Cryopreservation of ECAs/SEs

**Figure 8:**
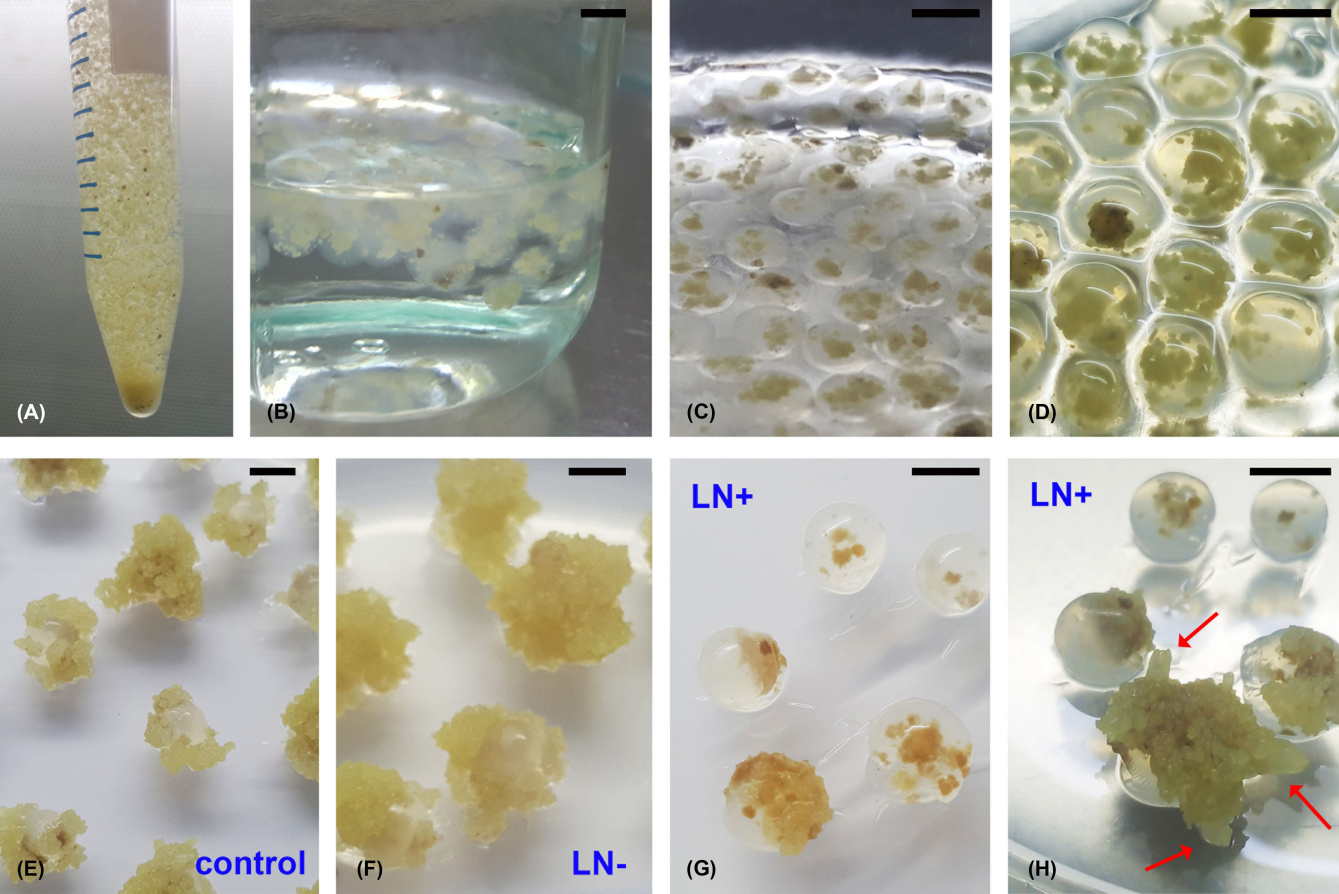
Cryopreservation of embryogenic cell suspensions of *A. flava* using the encapsulation-slow-freezing method. **(A)** Mixing the suspension with Na-alginate prior to bead preparation. **(B)** Formation of alginate beads in CaCl_2_-enriched liquid BM medium. **(C)** Cultivation of the alginate beads in liquid BM medium. **(D)** Beads after two days of incubation in liquid BM containing 0.9 M sorbitol. **(E, F)** Callus proliferation from alginate beads two weeks after encapsulation of control (untreated) beads **(E)** and beads that underwent cryopreservation pretreatment four weeks after thawing from −80°C (LN-) **(F)**. **(G, H)** Cryopreserved alginate beads four **(G)** and eight weeks **(H)** after thawing from LN (LN+). Red arrows indicate the formation of SEs. Scale bars = 5 mm.

**Figure 9:**
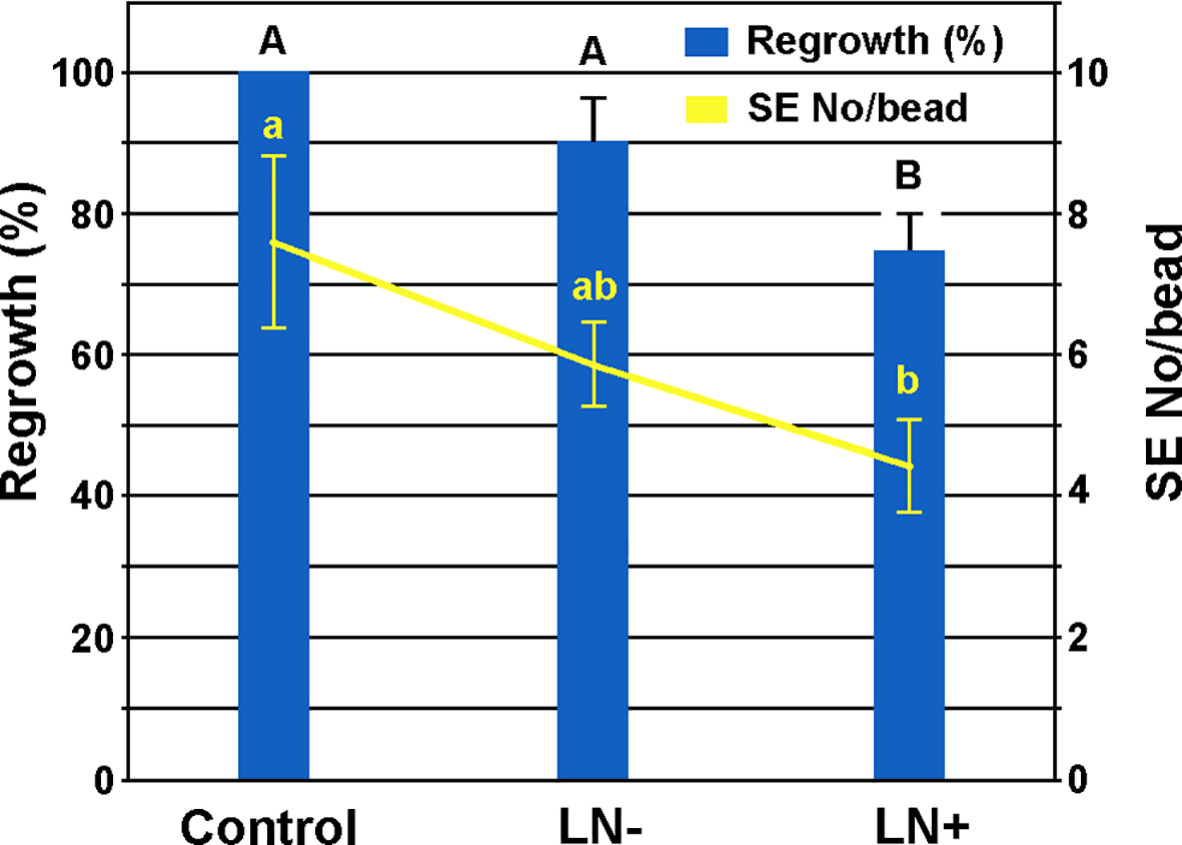
Regrowth and regeneration of SEs from ECAs of *A. flava* using encapsulation/slow-cooling method. Control: Non-cryopreserved encapsulated alginate beads; LN-: beads that underwent cryopreservation pretreatment and cooled for one hour in MrFrosty^®^ at –80°C; LN+ beads immersed in liquid nitrogen. The LN- and LN+ samples were thawed in a 40°C water bath for 3 min and the beads were then immersed in the solid 0.05/5 medium. ECAs growth and SEs number were recorded after eight weeks of cultivation.

Encapsulated ECAs/SEs of *A. flava* (**Figures 8A–D**) exhibited high regeneration potential even after cryopreservation (**Figures 8E–H**, **9**). After two weeks of culturing on solid 0.05/5 medium, newly formed embryogenic callus broke the alginate beads and enveloped the entire bead (**Figures 8E**, **9**). After eight weeks of cultivation, thawed beads from –80°C (LN-) showed a high regrowth frequency (over 90%) with no significant difference compared to the control (**Figures 8F**, **9**), while 75% of cryopreserved beads (LN+), thawed from LN, exhibited regrowth measured as covering the bead with new regenerated ECAs (**Figures 8G–H**, **9**). The average SEs number per bead was 7.60 ± 1.20 for the control beads, 5.86 ± 0.60 after thawing from −80°C (LN-), and 4.44 ± 0.66 after cryopreservation (LN+, **Figure 9**).

### 3.8 LC/MS profiling of SEs and ZEs ethanolic extracts

A comprehensive comparative LC/MS characterization of ethanolic extracts of ZEs and SEs at successive developmental stages revealed a total of 117 metabolites, including 12 benzoic acid derivatives, 8 cinnamic acid derivatives, 10 flavan-3-ol monomers and oligomers (procyanidins), 48 flavonoid glycosides, and 8 flavonoid aglycones (not belonging to flavan-3-ols), as well as 31 saponins (**Tables 4**, **5**; **Supplementary Table 1**). As expected, the metabolic profiles of SEs varied during development and in comparison to ZEs. The number of compounds detected increased steadily with SEs development, from 53 and 55 compounds in ECAs and GSEs, respectively, to 102 compounds detected in CSEs (**Table 5**). Thus, CSEs were the richest source of metabolites (**Tables 4**, **5**). Although CSE–2 and CSE–8 had the same number of compounds, they differed slightly in qualitative profiles, as CSE–2 contained more flavonoid glycosides and fewer saponins than CSE–8, and vice versa (**Tables 4**, **5**). ZEs contained only 86 compounds, but had the highest number and content of saponins (**Tables 4**, **5**). **Supplementary Table 1** lists MS details of the compounds and the references that confirm the previous presence of this compound in *Aesculus* species. Fifty eight compounds, not previously described in *Aesculus* species, were detected.

**Table 4:** LC/MS data of compounds identified in ethanolic extracts of ZEs and SEs of *A. flava* at SEs successive developmental stages.

No.	Compound	Retention time (min)	Molecular formula	Exact mass *m/z*	Present at developmental stage
1	Gallic acid hexoside	0.52	C1_3_H_15_O_10_	331.06758	TSE, LTSE, CSE-2, CSE-8
2	Gallic acid	0.63	C_7_H_5_O_5_	169.01478	ECA, GSE, HSE, TSE, LTSE, ZE
3	Dihydroxybenzoic acid hexoside	0.79	C_13_H_15_O_9_	315.07296	GSE, TSE, LTSE, CSE-2, CSE-8, ZE
4	Dihydroxybenzoic acid	0.79	C_7_H_5_O_4_	153.01975	ECA, CSE-2, CSE-8
5	Hydroxybenzoic acid hexoside	0.97	C_13_H_15_O_8_	299.07785	ECA, GSE, HSE, LTSE, CSE-2, CSE-8, ZE
6	Vanillic acid hexoside	1.28	C_14_H_17_O_9_	329.08853	CSE-2, CSE-8
7	Hydroxybenzoic acid isomer 1	2.52	C_7_H_5_O_3_	137.02480	ECA, GSE, HSE, TSE, LTSE, CSE-2
8	Hydroxybenzoyl-malic acid	5.14	C_11_H_9_O_7_	253.03582	ECA, GSE, HSE, TSE, LTSE, CSE-2, CSE-8, ZE
9	Hydroxybenzoic acid isomer 2	5.18	C_7_H_5_O_3_	137.02469	ECA, GSE, HSE, TSE, LTSE, CSE-2, CSE-8
10	Vanillic acid	5.41	C_8_H_7_O_4_	167.03527	ZE
11	Bis-hydroxybenzoic acid hexoside	6.03	C_20_H_19_O_10_	419.09845	ECA, GSE, HSE, TSE, LTSE, CSE-2, CSE-8, ZE
12	Benzoyl-malic acid	6.16	C_11_H_9_O_6_	237.04088	ECA, GSE, HSE, TSE, LTSE, CSE-2, CSE-8, ZE
13	Caffeic acid hexoside	4.64	C_15_H_17_O_9_	341.08792	ECA, HSE, TSE, LTSE, CSE-2
14	Ferulic acid hexoside	4.69	C_16_H_19_O_9_	355.10346	LTSE, CSE-8, ZE
15	p-Coumaric acid hexoside	4.89	C_15_H_17_O_8_	325.09351	ECA, GSE, HSE, TSE, LTSE, CSE-2, CSE-8
16	Caffeic acid	4.88	C_9_H_7_O_4_	179.03528	ECA, GSE, HSE, TSE, CSE-2, CSE-8, ZE
17	p-Coumaric acid pentosyl-hexoside	5.32	C_21_H_27_O_12_	471.15132	ECA, GSE, HSE, TSE, LTSE, CSE-2, CSE-8, ZE
18	p-Coumaric acid	5.63	C_9_H_7_O_3_	163.04035	ECA, GSE, HSE, TSE, LTSE, CSE-2, CSE-8, ZE
19	Ferulic acid	6.52	C_10_H_9_O_4_	193.05101	ECA, CSE-2
20	Methoxycinnamic acid	7.11	C_10_H_9_O_3_	177.05609	CSE-8, ZE
21	Epicatechin 7-O-hexoside	4.97	C_21_H_23_O_11_	451.12490	CSE-2, CSE-8, ZE
22	B type proanthocyanidin dimer [E)C-(E)C]	5.19	C_30_H_25_O_12_	577.13606	CSE-2, CSE-8, ZE
23	(Epi)catechin-(epi)catechin-(epi)catechin trimer B type	5.26	C_45_H_37_O_18_	865.20008	CSE-8
24	Epicatechin	5.33	C_15_H_13_O_6_	289.07231	ECA, GSE, HSE, TSE, LTSE, CSE-2, CSE-8, ZE
25	(Epi)afzelechin-(epi)catechin dimer B type	5.54	C_30_H_25_O_11_	561.14120	CSE-2, CSE-8, ZE
26	Procyanidin C1	5.58	C_45_H_37_O_18_	865.19870	CSE-2, CSE-8
27	Aesculitannin A	5.77	C_45_H_35_O_18_	863.18377	CSE-2, CSE-8, ZE
28	(Epi)afzelechin-(epi)catechin-(epi)catechin trimer B type	5.77	C_45_H_37_O_17_	849.20463	CSE-2, CSE-8
29	A type proanthocyanidin dimer 1 [E)GC-(E)C]	5.80	C_30_H_23_O_13_	591.11515	ZE
30	A type proanthocyanidin dimer 2 [E)C-(E)C]	6.13	C_30_H_23_O_12_	575.12041	ECA, GSE, HSE, TSE, CSE-2, CSE-8, ZE
31	Kaempferol glycoside derivative 1	5.11	C_33_H_39_O_20_	755.20514	ECA, GSE, HSE, TSE, LTSE, CSE-2
32	Quercetin 3-O-(6”-pentosyl)-hexoside-3’-O-hexoside	5.54	C_32_H_37_O_21_	757.18360	GSE, HSE, TSE, LTSE, CSE-2, CSE-8, ZE
33	Quercetin 3-O-(6”-rhamnosyl)-hexoside-3’-O-hexoside	5.57	C_33_H_39_O_21_	771.19992	ECA, GSE, HSE, TSE, LTSE, CSE-2, CSE-8, ZE
34	Quercetin glycoside derivative 1	5.62	C_33_H_39_O_20_	755.20502	ECA, GSE, HSE, TSE, LTSE, CSE-2, CSE-8, ZE
35	Taxifolin 3-O-hexoside	5.67	C_21_H_21_O_12_	465.10444	CSE-2, CSE-8, ZE
36	Isorhamnetin glycoside derivative 1	5.68	C_34_H_41_O_21_	785.21568	TSE, LTSE, CSE-2, CSE-8, ZE
37	Myricetin 3-O-hexoside	5.71	C_21_H_19_O_13_	479.08398	HSE, CSE-2, CSE-8
38	Quercetin 3,4’-di-O-hexoside	5.74	C_27_H_29_O_17_	625.14202	ECA, GSE, HSE, TSE, LTSE, CSE-2, CSE-8, ZE
39	Quercetin 3-O-(6”-pentosyl)-hexoside	5.79	C_26_H_27_O_16_	595.13119	ECA, GSE, HSE, TSE, LTSE, CSE-2, CSE-8, ZE
40	Kaempferol glycoside derivative 2	5.84	C_56_H_67_O_33_	1267.35794	LTSE, CSE-2, CSE-8
41	Kaempferol glycoside derivative 3	5.85	C_32_H_37_O_19_	725.19489	ECA, GSE, HSE, TSE, LTSE, CSE-2, CSE-8, ZE
42	Kaempferol glycoside derivative 4	5.85	C_33_H_39_O_19_	739.21046	ECA, GSE, HSE, TSE, LTSE, CSE-2, CSE-8, ZE
43	Quercetin 3-O-(6”-rhamnosyl)-hexoside (Rutin)	5.88	C_27_H_29_O_16_	609.14689	ECA, GSE, HSE, TSE, LTSE, CSE-2, CSE-8, ZE
44	Isorhamnetin glycoside derivative 2	5.90	C_34_H_41_O_20_	769.22016	ECA, GSE, HSE, TSE, LTSE, CSE-2, CSE-8, ZE
45	Isorhamnetin glycoside derivative 3	5.91	C_33_H_39_O_20_	755.20576	ECA, GSE, HSE, TSE, LTSE, CSE-2, CSE-8, ZE
46	Kaempferol 3-O-rhamnoside-7-O-pentoside	5.92	C_26_H_27_O_14_	563.14181	HSE, TSE, LTSE, CSE-2
47	Kaempferol 3-O-(2”-pentosyl-3”-hexosyl)-hexoside	5.96	C_32_H_37_O_20_	741.19006	GSE, HSE, TSE, LTSE, CSE-2, CSE-8, ZE
48	Eriodictyol 7-O-hexoside	6.00	C_21_H_21_O_11_	449.10906	ECA, GSE, CSE-2, CSE-8, ZE
49	Kaempferol 3-O-(2”-pentosyl)-hexoside (Leucoside)	6.01	C_26_H_27_O_15_	579.13656	ECA, GSE, HSE, TSE, LTSE, CSE-2, CSE-8, ZE
50	Quercetin 3-O-hexoside isomer 1	6.02	C_21_H_19_O_12_	463.08862	ECA, GSE, HSE, TSE, LTSE, CSE-2, CSE-8, ZE
51	Kaempferol 3-O-(4”-hexosyl)-rhamnoside (Multiflorin B)	6.03	C_27_H_29_O_15_	593.15203	ECA, GSE, HSE, TSE, LTSE, CSE-2, CSE-8, ZE
52	Kaempferol glycoside derivative 5	6.04	C_54_H_59_O_29_	1171.31571	HSE, TSE, LTSE, CSE-2, CSE-8
53	Kaempferol glycoside derivative 6	6.04	C_49_H_57_O_27_	1077.30990	GSE, HSE, TSE, LTSE, CSE-2, CSE-8
54	Kaempferol glycoside derivative 7	6.08	C_48_H_55_O_26_	1047.30025	ECA, GSE, HSE, TSE, LTSE, CSE-2, CSE-8, ZE
55	Isorhamnetin 3-O-(6”-pentosyl)-hexoside	6.08	C_27_H_29_O_16_	609.14685	ECA, GSE, HSE, TSE, CSE-2, CSE-8, ZE
56	Quercetin glycoside derivative 2	6.09	C_42_H_45_O_22_	901.24247	HSE, TSE, LTSE, CSE-2, CSE-8, ZE
57	Isorhamnetin 3-O-(6”-rhamnosyl)-hexoside	6.10	C_28_H_31_O_16_	623.16263	ECA, GSE, HSE, TSE, LTSE, CSE-2, CSE-8, ZE
58	Kaempferol 3,7-di-O-rhamnoside (Kaempferitrin)	6.10	C_27_H_29_O_14_	577.15729	ECA, GSE, HSE, TSE, LTSE, CSE-2, CSE-8, ZE
59	Quercetin glycoside derivative 3	6.19	C_62_H_69_O_33_	1341.37481	CSE-2, CSE-8
60	Naringenin 7-O-(6”-rhamnosyl)-hexoside	6.20	C_27_H_31_O_14_	579.17265	ECA, GSE, HSE, TSE, LTSE, CSE-2, CSE-8, ZE
61	Kaempferol glycoside derivative 8	6.22	C_57_H_61_O_29_	1209.33229	CSE-2, CSE-8, ZE
62	Kaempferol glycoside derivative 9	6.23	C_63_H_71_O_33_	1355.38951	CSE-2, CSE-8
63	Kaempferol glycoside derivative 10	6.24	C_42_H_45_O_21_	885.24720	ECA, GSE, HSE, TSE, LTSE, CSE-2, CSE-8, ZE
64	Kaempferol 3-O-hexoside	6.24	C_21_H_19_O_11_	447.09356	ECA, GSE, HSE, TSE, LTSE, CSE-2, CSE-8, ZE
65	Kaempferol 3-O-(2”-pentosyl)-rhamnoside	6.28	C_26_H_27_O_14_	563.14189	ECA, GSE, HSE, TSE, LTSE, CSE-2
66	Kaempferol glycoside derivative 11	6.31	C_62_H_69_O_32_	1325.37897	HSE, TSE, LTSE, CSE-2, CSE-8
67	Isorhamnetin 3-O-hexoside	6.32	C_22_H_21_O_12_	477.10430	ECA, GSE, HSE, TSE, LTSE, CSE-2, CSE-8, ZE
68	Naringenin 7-O-hexoside	6.32	C_21_H_21_O_10_	433.11429	ECA, GSE, HSE, TSE, LTSE, CSE-2, CSE-8, ZE
69	Kaempferol glycoside derivative 12	6.33	C_60_H_59_O_27_	1211.32723	TSE, LTSE, CSE-2, CSE-8, ZE
70	Kaempferol glycoside derivative 13	6.35	C_58_H_63_O_29_	1223.34737	HSE, TSE, LTSE, CSE-2, CSE-8
71	Kaempferol 3-O-(6′′-malonyl)-hexoside	6.40	C_24_H_21_O_14_	533.09480	ECA, GSE, HSE, TSE, LTSE, CSE-2, CSE-8
72	Kaempferol 3-O-[6’’’-acetyl-(4”-hexosyl)]-rhamnoside (Multiflorin A)	6.42	C_29_H_31_O_16_	635.16303	ECA, GSE, HSE, TSE, LTSE, CSE-2, CSE-8
73	Kaempferol 3-O-(6′′-acetyl)-hexoside	6.42	C_23_H_21_O_12_	489.10462	ECA, GSE, HSE, TSE, LTSE, CSE-2, CSE-8
74	Isorhamnetin 3-O-(6′′-acetyl)-hexoside	6.49	C_24_H_23_O_13_	519.11529	GSE, HSE, TSE, LTSE, CSE-2, CSE-8
75	Quercetin 3-O-hexoside isomer 2	6.49	C_21_H_19_O_12_	463.08864	ECA, GSE, HSE, TSE, LTSE, CSE-2, CSE-8, ZE
76	Isorhamnetin 3-O-rhamnoside	6.50	C_22_H_21_O_11_	461.10933	ECA, TSE, LTSE, CSE-2, CSE-8, ZE
77	Kaempferol 3-O-rhamnoside	6.54	C_21_H_19_O_10_	431.09876	ECA, GSE, HSE, TSE, LTSE, CSE-2, CSE-8, ZE
78	Apigenin 7-O-hexoside	6.71	C_21_H_19_O_10_	431.09887	TSE, LTSE
79	Eriodictyol	5.99	C_15_H_11_O_6_	287.05659	ECA, GSE, HSE, TSE, LTSE, CSE-2, CSE-8, ZE
80	Taxifolin	6.01	C_15_H_11_O_7_	303.05158	ECA, GSE, HSE, TSE, LTSE, CSE-2, CSE-8
81	Hesperetin	6.41	C_16_H_13_O_6_	301.07229	ECA, GSE, HSE, TSE, LTSE, CSE-2, CSE-8
82	Quercetin	6.98	C_15_H_9_O_7_	301.03611	ECA, GSE, HSE, TSE, LTSE, CSE-2, CSE-8, ZE
83	Naringenin	7.33	C_15_H_11_O_5_	271.06183	ECA, GSE, HSE, TSE, LTSE, CSE-2, CSE-8, ZE
84	Apigenin	7.36	C_15_H_9_O_5_	269.04617	GSE, HSE, TSE, LTSE, CSE-2, CSE-8, ZE
85	Kaempferol	7.42	C_15_H_9_O_6_	285.04110	ECA, GSE, HSE, TSE, LTSE, CSE-2, CSE-8, ZE
86	Isorhamnetin	7.50	C_16_H_11_O_7_	315.05193	ECA, GSE, CSE-2, CSE-8
87	Aescin derivative 1 (like escin IV)	6.49	C_52_H_81_O_24_	1089.51376	ZE
88	Aesculus saponin 1	6.66	C_42_H_67_O_17_	843.43886	CSE-8, ZE
89	Aesculiside N	6.74	C_49_H_77_O_22_	1017.49125	CSE-8, ZE
90	Aesculus saponin 2	7.57	C_58_H_89_O_26_	1201.56690	ZE
91	Aesculus saponin 3	7.71	C_55_H_91_O_29_	1215.56714	ZE
92	Assamicin VIII	7.86	C_47_H_73_O_18_	925.48164	CSE-2, CSE-8, ZE
93	Aesculioside IIc	8.03	C_52_H_81_O_21_	1041.52815	ZE
94	Aescin derivative 2 (like escin IVf)	8.04	C_53_H_83_O_23_	1087.53453	CSE-2, CSE-8, ZE
95	Aesculioside IIa or IIb	8.06	C_52_H_81_O_22_	1057.52357	CSE-8, ZE
96	Aesculus saponin 4	8.11	C_59_H_89_O_25_	1197.57031	ZE
97	Aescin derivative 3 (like escin V)	8.22	C_54_H_85_O_24_	1117.54410	CSE-2, CSE-8, ZE
98	Aescin derivative 4 (like isoescin IIb)	8.29	C_54_H_83_O_23_	1099.53448	CSE-2, CSE-8, ZE
99	Aesculus saponin 5	8.69	C_47_H_75_O_17_	911.50195	CSE-2, CSE-8, ZE
100	Aesculus saponin 6	8.38	C_51_H_81_O21	1029.52918	CSE-2, CSE-8, ZE)
101	Putranoside C	8.42	C_48_H_75_O_18_	939.49609	CSE-2, CSE-8, ZE
102	Aesculus saponin 7	8.44	C_49_H_75_O_19_	967.49190	CSE-2, CSE-8, ZE
103	Escin derivative 5 (like pavioside D)	8.59	C_54_H_83_O_22_	1083.53992	CSE-2, CSE-8, ZE
104	Aescin derivative 6 (like isoescin VIIa)	8.60	C_55_H_85_O_24_	1129.54482	CSE-2, CSE-8, ZE
105	Aesculioside G	8.75	C_56_H_87_O_24_	1143.56129	CSE-2, CSE-8, ZE
106	Aesculus saponin 8	8.87	C_52_H_79_O_20_	1023.51868	CSE-2, CSE-8, ZE
107	Aesculus saponin 9	8.88	C_51_H_77_O_19_	993.50775	CSE-2, CSE-8, ZE
108	Aesculioside IIIc	8.88	C_56_H_87_O_23_	1127.56764	CSE-2, CSE-8, ZE
109	Aesculus saponin 10	9.05	C_59_H_93_O_24_	1185.60902	CSE-2, CSE-8, ZE
110	Aesculiside P	9.06	C_57_H_87_O_23_	1139.56542	CSE-2, CSE-8, ZE
111	Aesculioside IV a or IVb	9.08	C_57_H_89_O_23_	1141.58014	CSE-2, CSE-8, ZE
112	Aesculus saponin 3	9.12	C_52_H_79_O_19_	1007.52320	CSE-2, CSE-8, ZE
113	Aesculus saponin 11	9.14	C_56_H_85_O_22_	1109.55457	CSE-2, CSE-8, ZE
114	Aesculus saponin 12	9.29	C_51_H_79_O_18_	979.52820	CSE-2, CSE-8, ZE
115	Aesculioside IV-23D1	9.38	C_57_H_87_O_22_	1123.57084	CSE-2, CSE-8, ZE
116	Aesculioside D or C	9.40	C_58_H_89_O_24_	1169.57676	CSE-2, CSE-8
117	Aesculus saponin 13	9.66	C_57_H_87_O_21_	1107.57593	CSE-2, CSE-8, ZE

ECA, embryogenic cell aggregates; GSE, globular SEs; HSE, heart-shaped SEs; TSE, torpedo SEs; LTSE, late torpedo SEs; CSE, cotyledonary SEs.

**Table 5:** Number of compounds detected in zygotic embryos (ZEs), embryogenic cell aggregates (ECAs) and somatic embryos (SEs) at globular (GSE), heart-shaped (HSE), early torpedo–shaped (TSE), late torpedo (LTSE) and cotyledonary (CSE) stage.

Developmental stage	Benzoic acid derivatives	Cinnamic acid derivatives	Flavan-3-ols and procyanidins	Flavonoid glycosides	Flavonoid aglycones	Saponins	Total
ECA	8	6	2	30	7	0	53
GSE	8	4	2	33	8	0	55
HSE	7	5	2	38	7	0	59
TSE	9	5	2	41	7	0	64
LTSE	9	5	1	41	7	0	63
CSE–2	10	6	8	47	8	23	102
CSE–8	9	6	9	44	8	26	102
ZE	7	5	7	32	5	30	86
Total	12	8	10	48	8	31	117

All SEs were cultured on solid 0.05/5 medium with 2% sucrose, except for CSE–8, which were cultured on 8% sucrose-supplemented medium.

**Figure 10:**
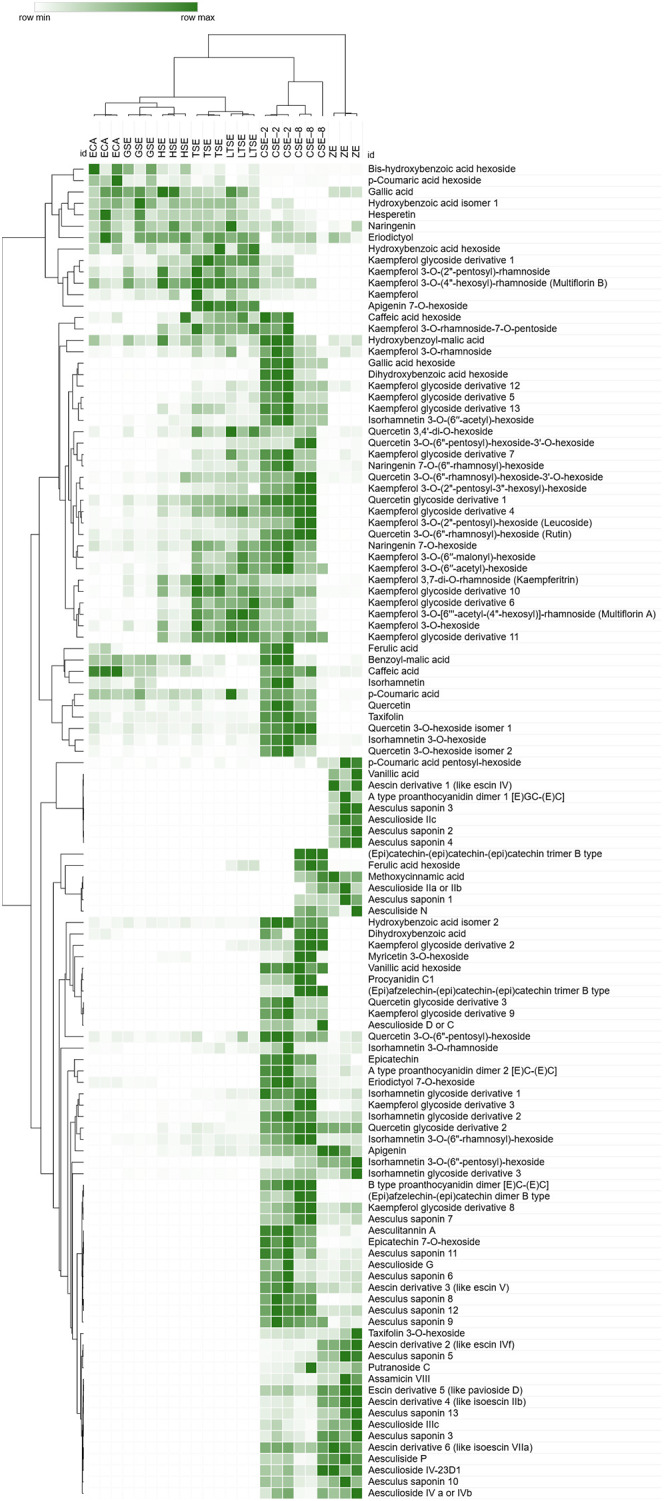
Heatmap of the relative abundance of 117 metabolites detected in embryogenic cell aggregates (ECAs), somatic embryos (SEs) at globular (GSE), heart-shaped (HSE), torpedo–shaped (TSE), late torpedo (LTSE), and cotyledonary (CSE-2 and CSE–8) stages, and zygotic embryos (ZEs) of *A. flava*. Relative abundance (defined as relative peak areas) was obtained from full-scan MS, using hierarchical cluster analysis (HCA) plots, constructed in Morpheus software, based on the Spearman method of cluster agglomeration, adopting the average linkage method. The compounds are shown in rows and the samples in columns. The green color gradient indicates the relative abundance of detected compounds, with darker shades representing higher abundance. Three biological replicates per ECA/SEs developmental stage were prepared.

Benzoic and cinnamic acid derivatives were much more abundant in CSEs, with only a few, such as gallic acid, detected in early SEs, and present in trace amounts in ZEs (**Tables 4**, **5**; **Figure 10**). SEs across all developmental stages contained higher amounts of flavonoids than ZEs (**Tables 4**, **5**; **Figure 10**). The flavonoid aglycones hesperetin, naringenin, eriodictyol and kaempferol were most abundant in early SEs up to the TSE stage, whereas quercetin, taxifolin and isorhamnetin were most abundant in CSEs, especially in CSE-2 (**Table 4**; **Figure 10**). In contrast, ZEs contained only five flavonoid aglycones in trace amounts (**Tables 4**, **5**), with apigenin being the most abundant, while taxifolin, hesperetin, and isorhamnetin were not detected (**Figure 10**). Of the 24 kaempferol glycosides detected in the present study, only 12 were present in trace amounts in ZEs. ZEs mainly contained quercetin glycosides (9 compounds) and isorhamnetin glycosides (7 compounds), the latter being predominant flavonoid glycosides. The number of flavonoid glycosides increased progressively from the ECA stage (30) to the CSE stage (47 and 44 in CSE–2 and CSE–8, respectively), while only 32 were detected in ZEs (**Tables 4**, **5**). Kaempferol and kaempferol glycosides were present in higher amounts, while quercetin and its glycosides were less abundant in young SEs than in CSEs or ZEs (**Table 4**; **Figure 10**). CSEs contained different flavonoid aglycones compared to younger SEs, with the highest content of quercetin, isorhamnetin and taxifolin. The amounts of flavonoids also differed between CSE–2 and CSE–8, with CSE–8 containing 21 of 24 kaempferol glycosides, confirming the CSEs as the richest source of flavonoids in the present study (**Tables 4**, **5**; **Figure 10**).

Only two flavan-3-ols and procyanidins, epicatechin and type A proanthocyanidin dimer 2, were present in trace amounts in the SEs up to the cotyledonary stage of development (**Tables 4**, **5**; **Figure 10**). CSEs contained the highest number (8 in CSE–2, 9 in CSE–8) and amount of flavan-3-ols/procyanidins, while 7 were detected in ZEs (**Tables 4**, **5**; **Figure 10**). Proanthocyanidin A1 dimer was the most abundant in ZEs, while it was not detected in the CSE–2 and CSE–8 samples (**Figure 10**). Epicatechin and type A proanthocyanidin 2 were more abundant in the CSE–2 than in CSE–8 samples, while others were more abundant in the CSE–8 samples (**Figure 10**). Therefore, the content of flavan-3-ols and procyanidins in CSEs more closely resembled that in ZEs than in earlier SE stages, reinforcing CSEs as the richest source of these compounds.

Aside from ZEs, saponins were only detected in CSEs, indicating that their synthesis in *A. flava* only begins in SEs at the most advanced stage of development (**Tables 4**, **5**). Of 31 saponins detected, 30 were found in ZEs, and 23 and 26 in CSE–2 and CSE–8, respectively (**Tables 4**, **5**). Twelve and ten new saponins were detected in ZEs and CSEs, respectively (**Table 4**; **Figure 10**). The metabolic profiles of saponins differed significantly between ZE and CSE samples: 19 saponins were present in higher amounts in ZEs, whereas nine saponins were more abundant in CSEs (**Figure 10**). In addition, six aescin derivatives, similar to those previously detected in other *Aesculus* species, were identified in ZEs and five in CSEs, with five being more abundant in ZEs and one in CSEs.

## 4 Discussion

### 4.1 The efficiency of embryogenic tissue induction: solid vs. liquid cultures

Woody plant species are generally more recalcitrant to *in vitro* regeneration than herbaceous species, making any enhancement in ET initiation frequency in these species of utmost importance (Andrade and Merkle, 2005; Bonga et al., 2010: Martínez et al., 2017). In the present study, ET initiation from the filament cultures of *A. flava* was increased about eight-fold by the application of liquid medium. The formation of PEMs was essentially the same in solid and liquid cultures, suggesting that the higher initiation frequency in liquid cultures is likely due to better nutrient and oxygen supply (von Arnold et al., 2002). Although necrosis occurred in both liquid and solid cultures, PEMs embedded in friable callus on solid medium were likely more exposed to toxic compounds released by necrotic tissue than PEMs in liquid cultures. Some of these compounds may have a deleterious effect on the embryogenic cells (Tang and Newton, 2004).

The application of a liquid medium or a liquid medium overlay over solid medium enhanced SEs initiation frequency in numerous woody species. In this way, the frequency of SEs initiation from megagametophytes of loblolly pine increased by 10% (Pullman and Skryabina, 2007), whereas embryogenic callus induction from immature ZEs of Chinese chestnut nearly doubled in liquid medium compared to solid medium (Li et al., 2022). Similarly, proliferation of friable callus and SEs regeneration were promoted in suspension cultures of date palms (Ibraheem et al., 2013). Accordingly, temporary immersion bioreactors significantly outperformed solid cultures in biomass production, SEs initiation, and secondary SEs regeneration than solid cultures in coconut, oil, and peach palms (Steinmacher et al., 2011; Gomes et al., 2016; Mu et al., 2024). The consistency of the medium not only affects regeneration efficiency but can even be a decisive factor for the *in vitro* regeneration pathway. For example, root segments of *Solanum khasianum* regenerated adventitious shoots on solid medium and SEs in liquid medium of the same composition (Pandey et al., 2018).

Furthermore, substantial variation in the frequency of ET initiation observed over a decade in this study suggests a strong influence of environmental factors on this process. Environmental conditions of plant material before and/or during explant isolation strongly influenced SEs initiation efficiency in *Pinus radiata* as well as the performance of SE–derived plants *ex vitro*, even months later (Montalbán et al., 2015). This suggests that stress conditions (e.g., thermal stress) likely induced epigenetic changes in plant material that promoted cellular reprogramming and prepared SE-derived plants for future stresses (Montalbán et al., 2015; Castander-Olarieta et al., 2020). However, cold storage or pre-heating of *A. flava* inflorescences or flower buds post-harvest and before filament isolation did not increase ET initiation frequency (data not shown).

Histological examination of ET revealed cells with typical meristematic features and intense mitotic activity, as observed in *A. hippocastanum* (Profumo et al., 1987) and other woody species (Corredoira et al., 2006, 2012, 2015; Deo et al., 2010). Highly vacuolated cells of variable shape were also observed at the periphery of the meristematic cell clusters, showing signs of cell degradation, which may lead to tissue fragmentation, as was observed in cork oak (Puigderrajols et al., 2001). Under the continued presence of 2,4-D, these clusters eventually undergo fragmentation as the result of auxin-mediated changes in cell walls, releasing smaller ECAs that perpetuate the cycle of growth and fragmentation (Halperin and Jensen, 1967; Puigderrajols et al., 2001). A relatively high proliferating capacity of *A. flava* suspension cultures is likely related to alternating cycles of growth and fragmentation. Such minute ECAs, composed of small meristematic cells and detached from the surrounding tissue in *A. hippocastanum* (Profumo et al., 1987) or from the embryo clusters in cork oak (Jiménez et al., 2013) are equivalent to ECAs in the present study.

The ET that formed from the friable callus in the present study was predominantly represented by PEMs, which correspond to the embryogenic nodular structures described in numerous woody plant species such as oaks, poplars, sweetgum, grapes, etc (Dai et al., 2004; Corredoira et al., 2006, 2012; Dhekney et al., 2011; Correia et al., 2012), while embryogenic callus was observed only rarely and briefly. In contrast to the results of the present study, *A. hippocastanum* primarily formed solid, white callus from different types of explants, which gave rise to two types of calli: a non–embryogenic friable callus or a yellowish, globular embryogenic callus (Dameri et al., 1986; Profumo et al., 1987; Capuana and Debergh, 1997). However, solid calli have not been observed in *A. flava* in the present or the previous study (Zdravković-Korać et al., 2019).

### 4.2 Initiation and maintenance of the suspension cultures

High callus friability is a prerequisite for cells to dissociate and form fine suspensions under agitation (Deo et al., 2010). Accordingly, friable calli induced from filaments of *A. flava* cultured on 1/10 medium were suitable for initiating embryogenic suspensions. In contrast, compact, solid calli obtained from different types of explants of *A. hippocastanum* and *A. carnea* (Radojević, 1988; Profumo et al., 1986; Gastaldo et al., 1994; Troch et al., 2009) were not suitable for suspension initiation (Dameri et al., 1986), as was also reported for other woody species (Kong et al., 2023). In *A. hippocastanum*, solid calli in liquid cultures never regenerated SEs and became non-embryogenic (Dameri et al., 1986), while friable calli formed only secondarily on solid calli and were considered non-embryogenic (Dameri et al., 1986; Profumo et al., 1987; Radojević, 1988). Suspension cultures can also be initiated by culturing clumps of SEs in liquid medium (Jiménez et al., 2013) and maintained by secondary somatic embryogenesis. Secondary somatic embryogenesis has been frequently observed in *Aesculus* species (Dameri et al., 1986; Profumo et al., 1987; Radojević, 1988; Kiss et al., 1992; Capuana and Debergh, 1997; Ćalić et al., 2005; Zdravković-Korać et al., 2008, 2019, 2022). Suspensions were typically initiated with cell aggregates of 40–800 μm (Andrade and Merkle, 2005; Jiménez et al., 2013). However, in the present study liquid cultures were preferably initiated using friable callus, prior to SEs initiation, to generate more embryogenic cell lines and provide a broader basis for the selection of lines suitable for further propagation.

Despite the suitability of the friable callus of *A. flava* for initiating suspension cultures, its dispersed cells could not proliferate under the conditions tested. Once the cell suspensions of *A. flava* were initiated, gradual browning and necrosis occurred, despite ample nutrient supply. As previously mentioned, necrotic tissue releases toxic compounds, such as phenolics, in explants’ response to stress from *in vitro* cultivation or cellular dedifferentiation (Alemanno et al., 2003; Tang and Newton, 2004; Reis et al., 2008). Tissue necrosis, its disruption, and the resulting isolation of cells are often associated with ET induction (Tulecke and McGranahan, 1985; Merkle et al., 1995; Corredoira et al., 2006, 2015; Reis et al., 2008; Dhekney et al., 2011; Jiménez et al., 2013). Necrotic tissue was surprisingly observed even in cork oak suspension cultures initiated from SEs clusters (Jiménez et al., 2013). In fact, necrosis is considered a potential cause of isolation of cells, which are then released from the influence of the surrounding tissue (Merkle et al., 1995). In addition, cells rich in phenolics can form a local barrier, isolating a small number of cells from the surrounding tissue (Reis et al., 2008). Notably, some phenolic compounds promote somatic embryogenesis in a dose-dependent manner, presumably due to their antioxidant effects, while others inhibit or have no effect on this process (Reis et al., 2008).

Liquid cultures of A. *flava* could only be maintained after the transition from cells that had emerged from a friable callus to isodiametric cells with dense cytoplasm and small vacuoles with a high proliferative capacity that eventually formed ECAs. ECAs were visible 4–8 weeks after liquid culture initiation and 8–12 weeks after filament isolation. This is consistent with the results obtained in white oak, where PEMs began to regenerate 7–8 weeks after culture initiation, with most responses occurring at 9–12 weeks (Corredoira et al., 2012). Similarly, suspension cultures of taro began to form cell aggregates after 3 months of cultivation (Deo et al., 2010).

### 4.3 Proliferation, maintenance and SEs regeneration from ECAs of different cell lines

Although all cultures originated from the same yellow buckeye tree and shared the same genotype, cell lines obtained from different explants in the present study differed considerably in proliferation and embryogenic capacity, indicating that these features are not solely genotype-dependent. Similar results were observed in our previous study (Zdravković-Korać et al., 2019) as well as in numerous woody plant species such as taro (Deo et al., 2010), cork oak (Jiménez et al., 2013), *Pinus thunbergii* (Sun et al., 2022) and Chinese chestnut (Li et al., 2022). In the present study, explants/suspensions with high PEM content produced highly proliferative cell lines that could be maintained for extended periods with high proliferation rates. This has also been observed in other woody plant species (Deo et al., 2010; Jiménez et al., 2013; Li et al., 2022). In contrast, in some cell lines, PEMs were only transiently observed as SEs rapidly progressed to the cotyledonary stage, usually causing these lines to perish after several subcultures, as reported for cork oak (Jiménez et al., 2013).

High variability in proliferation and embryogenic capacities observed among cell lines of the same genetic background can be attributed to epigenetic modifications that occur before and during *in vitro* cultivation. Such epigenetic mechanisms include regulation of gene expression through DNA methylation (Nic-Can et al., 2013), chromatin remodeling (Pérez et al., 2015a), and microRNA-mediated pathways (Wu et al., 2015), as demonstrated in *Coffea canephora*, *Quercus suber*, and *Citrus sinensis*. Cell morphology, micromorphology of SEs, and biochemical parameters, such as variations in soluble protein and sugar content, local imbalances in endogenous hormones, and the production of reactive oxygen species, can also play a decisive role in determining the proliferation rate of ET and the subsequent development of SEs (Pérez et al., 2015b; Salaj et al., 2019; Sun et al., 2022). In addition, external factors, such as cold storage, can influence embryogenic capacity. As previously mentioned, cold storage of *Pinus radiata* cones at 4°C for 1–4 months has been shown to positively affect the subsequent embryogenic potential of derived cell lines by increasing the number of SEs produced per gram of ET (Montalbán et al., 2015).

### 4.4 Size-fractionation of suspensions

Unlike animal and microbial cells, plant cells aggregate in culture, as daughter cells remain attached after cell division and adhere to each other due to increased excretion of polysaccharides and high cell density, particularly in the late exponential growth phase (Mavituna and Park, 1987; Santos et al., 2016). In large plant cell aggregates, central cells often experience restricted nutrient and oxygen availability (Santos et al., 2016), necessitating filtration to remove these aggregates and sustain suspension culture viability. In the present study, we observed necrotic zone in the center of each ECA, with brown clumps appearing after prolonged cultivation or without filtration of the suspension. As cell clumps enlarge, they gradually lose their proliferative capacity, age and eventually turn brown (Kong et al., 2014). Therefore, regular medium renewal and suspension filtration are critical to sustain proliferation and maintain a predominance of small and medium–sized cell aggregates in suspension cultures (Martínez et al., 2023).

Furthermore, suspension fractionation promotes synchronized SEs development and facilitates SEs handling for further use. In the present study, suspension fractionation enabled the separation of SEs by developmental stage and thus facilitated their selection for further use. In agreement with this, size-fractionated suspension cultures of American chestnut collected on a nylon mesh and placed over semi-solid medium produced synchronized populations of embryos with higher conversion frequencies than those of SEs cultured continuously on semi-solid medium (Andrade and Merkle, 2005), while cultivation in a liquid medium and sieving accelerated CSEs development in Chinese chestnut by three weeks (Li et al., 2022).

In the present study, the fraction of the smallest SEs (≤ 0.6 mm) had the highest proliferation rate. This fraction contained approximately 50% GSEs but very few CSEs, rendering it optimal for proliferation when high multiplication rates and rapid turnover are needed. This is consistent with the results of previous studies showing that the SEs of *Aesculus* sp. exhibit the highest potential for secondary somatic embryogenesis at earlier developmental stages (Kiss et al., 1992; Zdravković-Korać et al., 2008, 2019). In agreement with our findings, a 41–180 μm size fraction of cork oak, comprising detached cells and embryogenic masses, contained 60% of SEs sized 41–180 μm after four weeks of cultivation, while only 18% of SEs exceeded 800 μm (Jiménez et al., 2013). Although the 0.9–2.38 mm size fraction of SEs still showed a high proliferation rate in the present study, it contained predominantly late-stage SEs (~66% of TSEs + LTSEs + CSEs), making this fraction suitable for advancing SEs to the CSEs stage and producing somatic seedlings. At this stage, secondary somatic embryogenesis gradually decreases (Kiss et al., 1992; Zdravković-Korać et al., 2008, 2019). The CSEs of *A. flava* obtained in the present study showed a high germination rate of over 80% (Zdravković-Korać et al., 2019).

In the present study, an acceptable percentage of abnormal SEs was observed. Morphological abnormalities in SEs have been reported in numerous plant species (Corredoira et al., 2015), including *A. hippocastanum* and *A. carnea* (Dameri et al., 1986; Radojević et al., 1989; Radojević, 1988; Jörgensen, 1989; Capuana and Debergh, 1997). In some cases, the proportion of abnormal SEs was quite high; for example, in tamarillo abnormal SEs outnumbered normal ones by threefold, yet most were able to germinate and develop into viable plants (Correia et al., 2012).

In the present study, SEs cultured on 0.05/5 medium with 2–10% sucrose continued to increase in size, leading to greater biomass and dry matter content. Treatment with 8% sucrose was optimal for both biomass and dry matter accumulation. Similarly, 6% sucrose enhanced dry matter content in cell suspensions of *Satureja khuzistanica* (Sahraroo et al., 2018) and immature embryos of oil palm (Aberlenc-Bertossi et al., 2003).

The high efficiency achieved in the present study for initiation, proliferation, and SEs regeneration, combined with the strong germination capacity of the resulting CSEs, demonstrates promising potential for the ornamental propagation of *A. flava*. However, further optimization of the protocol is still required to ensure consistent and robust performance across different *A. flava* clones. Additionally, for reliable clonal propagation, an efficient protocol for the conversion of somatic embryos into plantlets needs to be established, followed by confirmation of clonal fidelity, successful acclimatization, and evaluation of field performance of the resulting somatic plants.

Even the most proliferative embryogenic cell suspensions gradually decline in proliferation and can be maintained for approximately six months (von Arnold et al., 2002; Deo et al., 2010). To preserve the optimal proliferative capacity of cell lines, ECAs should be cryopreserved once suspensions reach high, sustained proliferation (von Arnold et al., 2002).

### 4.5 Cryopreservation of ECAs/SEs

Cryopreservation of *A. flava* ECAs by encapsulation and slow cooling was successful in the present study, achieving 75% regrowth of the ECAs and subsequent reconstitution of the embryogenic suspension lines. Comparable recovery efficiencies have been reported for some other woody species (Corredoira et al., 2004; Martínez et al., 2022). In addition, the use of MrFrosty containers with isopropanol and a –80°C freezer in the present study proved to be a cost-effective and user-friendly alternative to specialized controlled-rate freezers, which can be very expensive (Schumacher et al., 2015). In general, three main methods have evolved for the cryopreservation of plant cell cultures: slow freezing, vitrification, and encapsulation, each with multiple variations (Nausch and Buyer, 2021). However, slow freezing remains the most efficient cryopreservation method, achieving 20–100% recovery after thawing from liquid nitrogen (Nausch and Buyer, 2021).

ET and SEs of *A. hippocastanum* were successfully cryopreserved using desiccation and vitrification methods, with high recovery rates of 48% (Jekkel et al., 1998) and 75% (Lambardi et al., 2005). For cryopreservation, SEs at globular to cotyledonary stages were used, yielding ET regrowth of 32.5%–46% for GSEs and 75% for TSEs (Jekkel et al., 1998; Lambardi et al., 2005). Jekkel et al. (1998) found that pre-treatment with 0.75 μM abscisic acid followed by 4-h air-drying to 13% water content and subsequent direct immersion in liquid nitrogen was even more efficient than slow- or rapid-cooling protocols using cryoprotectants (0.5 M DMSO + 0.5 M glycerol + 1 M sucrose). However, Lambardi et al. (2005) achieved higher recovery rates with a single-step vitrification procedure. After a five-day pre-culture at 4°C and a 60-min osmotic dehydration with Plant Vitrification Solution 2, the embryogenic potential of embryogenic cell masses containing TSEs was easily restored after thawing from liquid nitrogen, with optimized thawing procedure enabling a recovery of 94% TSEs (Lambardi et al., 2005). This confirms that the vitrification procedure works better with small, uniform explants and is less efficient with cell suspensions. In our study, we used encapsulation procedure which stabilizes cell suspensions by immobilizing cells within beads, beneficial when dealing with heterogeneous cell populations and aggregates in suspensions. Furthermore, the vitrification procedure is more complex, technique-dependent, and requires rapid manipulation and precise timing. The results of our study showed that the slow cooling method, with implementation of a simple device like Mr Frosty for controlled cooling combined with immobilization of cells in alginate, has great potential for long-term cryopreservation of embryogenic cell suspensions in laboratories with limited infrastructure.

### 4.6 LC/MS profiling of SEs and ZEs ethanolic extracts

The present study identified 117 compounds in the ethanolic extracts of SEs and ZEs of *A. flava*. To our knowledge, no prior study has provided a comprehensive metabolic profile of SEs during their development in any *Aesculus* species. For *A. flava*, only the chemical composition of immature fruits, flowers and pedicels has been analyzed (Green et al., 2021). The only relevant study for comparing secondary metabolite profiles during SEs development is the study by Kędzierski et al. (2016), which analyzed secondary metabolite content in the seeds of *A. hippocastanum* during their maturation, in the period of 7–21 weeks after anthesis. However, comparing these findings is challenging, as Kędzierski et al. (2016) did not track ZE developmental stages, although it can be assumed that most ZEs were already in the cotyledonary stage by week seven after anthesis (List and Steward, 1965).

SEs of *A. flava* exhibited a high flavonoid content, which increased steadily from the globular to the cotyledonary stage of development. In younger SEs (up to the TSE), kaempferol glycosides were predominant, but their levels decreased as SEs developed, with quercetin, taxifolin, isorhamnetin, and their glycosides becoming most abundant in CSEs. In contrast, kaempferol content in *A. hippocastanum* increased during seed maturation from the 7th to the 19th week after anthesis (Kędzierski et al., 2016). In *A. flava* ZEs (22 weeks after anthesis), flavonoid aglycones, with the exception of apigenin, were present only in trace amounts, with quercetin and isorhamnetin glycosides being the most abundant. Similarly, quercetin glycosides were predominant in the seeds and seed pulp of *A. hippocastanum* (Kapusta et al., 2007; Dridi et al., 2023) and the peeled seeds of *A. turbinata* (Kimura et al., 2017), as well as in the leaves of *A. hippocastanum*, *A. carnea* and *A. chinensis* (Oszmiański et al., 2014; Kimura et al., 2017; Yin et al., 2022) and the flowers of *A. chinensis* (Yin et al., 2022). Thirty-seven flavonoids were detected in *A. flava* ZEs, compared to 13 in *A. hippocastanum* seeds and 10 in its seed pulp (Kapusta et al., 2007; Dridi et al., 2023).

Although both CSE-2 and CSE-8 were rich in flavonoids, their flavonoid content differed, indicating not only a role of sucrose in flavonoid synthesis, but also that flavonoid levels can be readily manipulated. Literature data suggest varied effects of sucrose on flavonoid production. A high sucrose concentration (5%) was required to enhance flavonoid production in cell cultures of *Glycyrrhiza inflata* (Yang et al., 2009), while lower levels of 2-2.5% were optimal for cell suspensions of *Prunella vulgaris* (Fazal et al., 2016), and only 1% was sufficient for suspension cultures of *Morinda citrifolia* (Baque et al., 2012). Suspension cultures are considered a promising approach for flavonoid production (Mamdouh and Smetanska, 2022; Sui et al., 2022).

Procyanidin A2 content peaked in *A. hippocastanum* seeds 12–14 weeks after anthesis (Kędzierski et al., 2016). This compound was also detected in *A. flava* SEs across all developmental stages, with the highest levels in CSE-2 samples. In contrast, procyanidin A2 was present only in trace amounts in ZEs of *A. flava*, where the proanthocyanidin A1 dimer was predominant. Procyanidins were also prominent in the immature fruits of all 18 *Aesculus* species examined (Green et al., 2021).

In the present study, saponins, including aescin, were detected only in CSEs and ZEs. Consistent with our findings, Kędzierski et al. (2016) reported trace amounts of aescin in horse chestnut seeds from the 12th week after anthesis, with both qualitative and quantitative increases during seed development, peaking at 19 weeks post-anthesis. In contrast, aescin was detected in non-embryogenic and embryogenic calli and embryoids of horse chestnut (developmental stages unspecified) (Profumo et al., 1991, 1994), as well as in GSEs and CSEs of *A. hippocastanum* (Ćalić et al., 2010). Total aescin content was significantly higher in CSEs than in GSEs of horse chestnut (Ćalić et al., 2010), and was notably elevated in CSEs cultured on media supplemented with plant growth regulators (PGRs), such as 2,4-D, α-naphthaleneacetic acid, indole-3-butyric acid, Kin or 6-benzylaminopurine compared to PGR-free medium (Profumo et al., 1992; Ćalić et al., 2010). The origin of the SEs (leaves, cotyledons, or stem segments) did not affect aescin content (Profumo et al., 1991, 1994). Discrepancies between the findings of the present study and those of the studies mentioned above may be attributed to the use of a more sensitive analytical method in this study.

Thirty saponins were identified in ZEs of *A. flava*. Each *Aesculus* species contains numerous triterpenoids; for example, 33–35 triterpenoid saponins were detected in seed extracts of *A. chinensis* Bunge, *A. chinensis* var. chekiangensis, *A. hippocastanum*, and *A. wilsonii* (Zhang et al., 2020; Wang et al., 2023). Some of these compounds are species-specific and could serve as markers to differentiate *Aesculus* species. However, a comparative metabolomic study of immature fruits, flowers, and pedicels across 18 *Aesculus* species, including *A. flava*, revealed greater chemical differences among plant organs than among species (Green et al., 2021).

In summary, metabolic profiling of immature seeds of *Aesculus* species has shown that valuable compounds, such as aescin, kaempferol, and procyanidins, are present at highest levels in fruits and seeds well before abscision (Kędzierski et al., 2016; Green et al., 2021), thus raising questions about their availability. By contrast, the production of ECAs/SEs in suspension cultures ensures year-round availability of plant material for extraction. Further research is needed to optimize conditions for the production of targeted specialized metabolites and to confirm their biological activity. Optimizing cell growth in suspensions could enhance sustainable production of high-quality biomolecules (Aguilar-Camacho et al., 2023). Elicitation of plant suspension cultures with natural and synthetic molecules can further increase specialized metabolite yields (Arya et al., 2020, 2022; Sui et al., 2022; Murthy et al., 2024). It would be interesting to characterize the metabolite profile of acclimatized somatic plants, evaluate its stability across independent cell lines, and compare it with that of *A. flava* seedlings.

## 5 Conclusions

The present study addresses the challenge of low and variable embryogenic tissue initiation in *A. flava*, achieving a reliable 70–90% initiation efficiency using optimized liquid culture systems. For the first time, we have established embryogenic suspensions in *Aesculus* sp. with sustained proliferation and efficient SEs regeneration, advancing embryos to the cotyledonary stage with high germination rates. Our work also demonstrates successful cryopreservation of embryogenic tissue, with 75% regrowth after liquid nitrogen storage, ensuring long-term preservation. Metabolic profiling provided valuable insights into dynamic metabolic alterations during embryo development and revealed that cotyledonary-stage embryos are rich in flavonoids, procyanidins, and saponins. The production of ECAs/SEs in suspension cultures ensures year-round availability of plant material and may represent a promising source for extracting commercially relevant, contaminant-free metabolites for the pharmaceutical, cosmetic and food industries. However, further optimization of the protocol is still needed to achieve consistent performance across different *A. flava* clones/genotypes. In addition, comprehensive validation of metabolite yield, purity, and biological activity is required, as heavy metal analysis and bioactivity testing were not conducted in the present study.

## Data Availability

The raw data supporting the conclusions of this article will be made available by the authors, without undue reservation.
