# Supplementary material for: Enhanced initiation of somatic embryos in suspension cultures of Aesculus flava and metabolic profile of zygotic embryos and somatic embryos during their development
Source: Front Plant Sci. 2026 Jan 14;16:1736161. doi: 10.3389/fpls.2025.1736161 (PMC12847295; doi:10.3389/fpls.2025.1736161)
Supplement: Supplementary file 1 [file Table1.docx]

**Supplementary Material**

**Supplementary Table 1** LC/MS data of compounds identified in ethanolic extracts of ZEs and SEs of *A. flava*.

| **No.** | | **Compound** | **Retention time (min)** | **Molecular formula** | **Calculated mass**  ***m/z*** | **Exact mass**  ***m/z*** | **ppm** | **MS Fragments**  **(% Base peak)** | **References** |
| --- | --- | --- | --- | --- | --- | --- | --- | --- | --- |
| **Benzoic acid derivatives** | | | | | | | | | |
| **1** | Gallic acid hexoside | | 0.52 | C1_3_H_15_O_10_ | 331.06713 | 331.06758 | -1.36 | 125.02464(49), 168.00665(83), 169.01422(8), 313.05713(22), **331.06757**(100) | / |
| **2** | Gallic acid | | 0.63 | C_7_H_5_O_5_ | 169.01425 | 169.01478 | -3.13 | **125.02460**(100), 169.01477(49) | Owczarek et al. 2021 |
| **3** | Dihydroxybenzoic acid hexoside | | 0.79 | C_13_H_15_O_9_ | 315.07216 | 315.07296 | -2.56 | 108.02193(41), 109.02894(19), 152.01180(83), **153.01585**(100) | Oszmiański et al. 2015 |
| **4** | Dihydroxybenzoic acid | | 0.79 | C_7_H_5_O_4_ | 153.01933 | 153.01975 | -2.73 | 109.03014(13), **153.01967**(100) | Oszmiański et al. 2015 |
| **5** | Hydroxybenzoic acid hexoside | | 0.97 | C_13_H_15_O_8_ | 299.07724 | 299.07785 | -2.04 | 101.02463(30), 113.02467(23), 119.03526(29), **137.02473**(100), 179.03539(50), 239.05679(18) | / |
| **6** | Vanillic acid hexoside | | 1.28 | C_14_H_17_O_9_ | 329.08781 | 329.08853 | -2.21 | 101.02457(38), 113.02469(26), 119.03519(32), 123.04536(16), 152.01141(6), **167.03529**(100) | / |
| **7** | Hydroxybenzoic acid isomer 1 | | 2.52 | C_7_H_5_O_3_ | 137.02442 | 137.02480 | -2.81 | **93.03481**(100), 137.02480(27) | Owczarek et al. 2021 |
| **8** | Hydroxybenzoyl-malic acid | | 5.14 | C_11_H_9_O_7_ | 253.03538 | 253.03582 | -1.77 | 71.01403(16), 93.03477(17), 115.00396(10), **137.02472**(100) | / |
| **9** | Hydroxybenzoic acid isomer 2 | | 5.18 | C_7_H_5_O_3_ | 137.02442 | 137.02469 | -1.96 | **93.03478**(100), 137.02483(83) | Owczarek et al. 2021 |
| **10** | Vanillic acid | | 5.41 | C_8_H_7_O_4_ | 167.03498 | 167.03527 | -1.74 | 108.02196(37), **123.04551**(100), 152.01198(50), 167.03566(36) | Sarikurkcu et al. 2020 |
| **11** | Bis-hydroxybenzoic acid hexoside | | 6.03 | C_20_H_19_O_10_ | 419.09837 | 419.09845 | -0.18 | 93.03467(11), **137.02454**(100), 281.06693(25) | / |
| **12** | Benzoyl-malic acid | | 6.16 | C_11_H_9_O_6_ | 237.04046 | 237.04088 | -1.75 | 71.01401(20), 115.00392(10), **121.02974**(100) | / |
| **Cinnamic acid derivatives** | | | | | | | | | |
| **13** | Caffeic acid hexoside | | 4.64 | C_15_H_17_O_9_ | 341.08781 | 341.08792 | -0.33 | 135.04543(15), **179.03535**(100) | Dias et al. 2022 |
| **14** | Ferulic acid hexoside | | 4.69 | C_16_H_19_O_9_ | 355.10346 | 355.10346 | -0.01 | 134.03748(65), 149.06111(78), 178.02774(26), **193.05109**(100) | / |
| **15** | p-Coumaric acid hexoside | | 4.89 | C_15_H_17_O_8_ | 325.09289 | 325.09351 | -1.89 | 119.05046(5), **145.02979**(100), 163.04047(10) | / |
| **16** | Caffeic acid | | 4.88 | C_9_H_7_O_4_ | 179.03498 | 179.03528 | -1.66 | **135.04527**(100) | Owczarek et al. 2021 |
| **17** | p-Coumaric acid pentosyl-hexoside | | 5.32 | C_21_H_27_O_12_ | 471.15080 | 471.15132 | -1.11 | 119.05047(13), **145.02979**(100), 163.04044(56) | / |
| **18** | p-Coumaric acid | | 5.63 | C_9_H_7_O_3_ | 163.04007 | 163.04035 | -1.75 | **119.05052**(100), 163.04051(12) | Owczarek et al. 2021 |
| **19** | Ferulic acid | | 6.52 | C_10_H_9_O_4_ | 193.05063 | 193.05101 | -1.98 | 134.03778(30), 161.02466(19), 178.02724(6), **193.05101**(100) | Bielarska et al. 2022 |
| **20** | Methoxycinnamic acid | | 7.11 | C_10_H_9_O_3_ | 177.05572 | 177.05609 | -2.11 | 118.04271(35), 145.02992(33), 162.03247(10), **177.05612**(100) | / |
| **Flavan-3-ols and procyanidins** | | | | | | | | | |
| **21** | Epicatechin 7-O-hexoside | | 4.97 | C_21_H_23_O_11_ | 451.12459 | 451.12490 | -0.70 | 125.02475(12), 179.03537(10), 203.07179(13), 205.05109(13), 245.08244(47), **289.07233**(100) | / |
| **22** | B type proanthocyanidin dimer [E)C-(E)C] | | 5.19 | C_30_H_25_O_12_ | 577.13515 | 577.13606 | -1.58 | 125.02470(35), 179.03548(14), 203.07184(20), 245.08240(60), **289.07245**(100), 409.08307(14) | Oszmiański et al. 2015 |
| **23** | (Epi)catechin-(epi)catechin-(epi)catechin trimer B type | | 5.26 | C_45_H_37_O_18_ | 865.19855 | 865.20008 | -1.77 | **125.02470**(100), 161.02483(30), 243.03047(24), 287.0567(18), 289.07245(46), 407.07721(50) | Santos-Buelga et al. 1995 |
| **24** | Epicatechin | | 5.33 | C_15_H_13_O_6_ | 289.07176 | 289.07231 | -1.89 | 151.04045(39), 179.03549(33), 203.07178(76), 205.05109(50), 221.08238(26), **245.08247**(100) | Oszmiański et al. 2015 |
| **25** | (Epi)afzelechin-(epi)catechin dimer B type | | 5.54 | C_30_H_25_O_11_ | 561.14024 | 561.14120 | -1.71 | 125.02471(26), 203.07176(13), 205.05075(11), 245.08243(42), **289.07239**(100), 407.07785(16) | / |
| **26** | Procyanidin C1 | | 5.58 | C_45_H_37_O_18_ | 865.19854 | 865.19870 | -0.18 | **125.02473**(100), 161.02481(29), 243.03046(23), 287.05664(18), 289.07248(48), 407.07721(50) | Morimoto et al. 1987 |
| **27** | Aesculitannin A | | 5.77 | C_45_H_35_O_18_ | 863.18289 | 863.18377 | -1.02 | **125.02475**(100), 161.02483(35), 285.04074(20), 287.05551(19), 289.07242(57), 407.07742(53) | Morimoto et al. 1987 |
| **28** | (Epi)afzelechin-(epi)catechin-(epi)catechin trimer B type | | 5.77 | C_45_H_37_O_17_ | 849.20362 | 849.20463 | -1.18 | **125.02473**(100), 161.02493(26), 245.08267(12), 271.06125(15), 289.07266(50), 407.07755(42) | / |
| **29** | A type proanthocyanidin dimer 1 [E)GC-(E)C] | | 5.80 | C_30_H_23_O_13_ | 591.11442 | 591.11515 | -1.24 | 125.02467(55), 175.00401(34), 285.04077(40), 289.07230(32), **301.03592**(100), 407.07751(35) | / |
| **30** | A type proanthocyanidin dimer 2 [E)C-(E)C] | | 6.13 | C_30_H_23_O_12_ | 575.11950 | 575.12041 | -1.58 | 245.08223(14), **285.04092**(100), 289.07227(41), 407.07724(18), 423.07230(12), 539.09875(30) | Owczarek et al. 2021 |
| **Flavonoid glycosides** | | | | | | | | | |
| **31** | Kaempferol glycoside derivative 1 | | 5.11 | C_33_H_39_O_20_ | 755.20402 | 755.20514 | -1.48 | **284.03165**(100), 285.03598(80), 446.08609(30), 447.08929(35) | / |
| **32** | Quercetin 3-O-(6"-pentosyl)-hexoside-3'-O-hexoside | | 5.54 | C_32_H_37_O_21_ | 757.18328 | 757.18360 | -0.42 | 178.99893(3), **300.02805**(100), 301.03537(15), 462.08087(17), **595.13202**(35) | Kimura et al. 2017 |
| **33** | Quercetin 3-O-(6"-rhamnosyl)-hexoside-3'-O-hexoside | | 5.57 | C_33_H_39_O_21_ | 771.19893 | 771.19992 | -1.28 | 178.99870(3), **300.02808**(100), 301.03537(11), 462.08084(17), 609.14758(34) | / |
| **34** | Quercetin glycoside derivative 1 | | 5.62 | C_33_H_39_O_20_ | 755.20402 | 755.20502 | -1.33 | 300.02817(12), **301.03592**(100), 446.08597(39), 447.09363(50), 609.14709(14) | / |
| **35** | Taxifolin 3-O-hexoside | | 5.67 | C_21_H_21_O_12_ | 465.10334 | 465.10444 | -2.37 | **125.02467**(100), 259.06177(30), 275.05667(30), 285.04099(60), 303.05057(6), 437.10941(33) | / |
| **36** | Isorhamnetin glycoside derivative 1 | | 5.68 | C_34_H_41_O_21_ | 785.21458 | 785.21568 | -1.40 | 300.02786(45), **315.05151**(100), 461.07214(6), 477.10422(18), 623.16321(10) | / |
| **37** | Myricetin 3-O-hexoside | | 5.71 | C_21_H_19_O_13_ | 479.08311 | 479.08398 | -1.80 | 125.02476(5), 153.01967(11), 178.99907(3), **316.02298**(100), 317.02905(12) | Dudek-Makuch et al. 2019 |
| **38** | Quercetin 3,4'-di-O-hexoside | | 5.74 | C_27_H_29_O_17_ | 625.14102 | 625.14202 | -1.59 | 221.04597(56), 300.02808(51), **301.03595**(100), 462.08075(10), 463.08887(38) | Deyab et al. 2022 |
| **39** | Quercetin 3-O-(6"-pentosyl)-hexoside | | 5.79 | C_26_H_27_O_16_ | 595.13046 | 595.13119 | -1.22 | **300.02829**(100), 301.03433(7) | Kimura et al. 2017 |
| **40** | Kaempferol glycoside derivative 2 | | 5.84 | C_56_H_67_O_33_ | 1267.35701 | 1267.35794 | -0.73 | **284.03305**(100), 285.04041(18), 739.21021(52), 769.22131(7) | / |
| **41** | Kaempferol glycoside derivative 3 | | 5.85 | C_32_H_37_O_19_ | 725.19356 | 725.19489 | -1.83 | 255.03036(3), **284.03314**(100), 285.04062(20), 575.14221(4), 725.19489(10) | / |
| **42** | Kaempferol glycoside derivative 4 | | 5.85 | C_33_H_39_O_19_ | 739.20911 | 739.21046 | -1.82 | 255.03020(3), **284.03299**(100), 285.04022(15) | / |
| **43** | Quercetin 3-O-(6"-rhamnosyl)-hexoside (Rutin) | | 5.88 | C_27_H_29_O_16_ | 609.14611 | 609.14689 | -1.28 | 151.00392(3), 178.99898(3), **300.02808**(100), 301.03571(46), 609.14752(15) | Oszmiański et al. 2015 |
| **44** | Isorhamnetin glycoside derivative 2 | | 5.90 | C_34_H_41_O_20_ | 769.21967 | 769.22016 | -0.64 | 300.02841(8), **315.05167**(100) | / |
| **45** | Isorhamnetin glycoside derivative 3 | | 5.91 | C_33_H_39_O_20_ | 755.20357 | 755.20576 | -2.90 | 271.02505(9), 299.02026(90), 300.02795(71), **314.04382**(100), 315.05161(97), 755.20575(40) | / |
| **46** | Kaempferol 3-O-rhamnoside-7-O-pentoside | | 5.92 | C_26_H_27_O_14_ | 563.14062 | 563.14181 | -2.12 | 283.02548(80), 284.03207(6), 285.04108(56), 417.08154(7), **430.09097**(100), 431.09790(21) | / |
| **47** | Kaempferol 3-O-(2"-pentosyl-3"-hexosyl)-hexoside | | 5.96 | C_32_H_37_O_20_ | 741.18837 | 741.19006 | -2.28 | 255.03040(4), **284.03308**(100), 285.04062(39) | Kapusta et al. 2007 |
| **48** | Eriodictyol 7-O-hexoside | | 6.00 | C_21_H_21_O_11_ | 449.10894 | 449.10906 | -0.27 | 125.02482(4), 135.04527(30), **151.00383**(100), 175.00372(10), 287.056520(53) | / |
| **49** | Kaempferol 3-O-(2"-pentosyl)-hexoside (Leucoside) | | 6.01 | C_26_H_27_O_15_ | 579.13554 | 579.13656 | -1.76 | **284.03293**(100), 285.04047(17), 579.13654(4) | Wei et al. 2004 |
| **50** | Quercetin 3-O-hexoside isomer 1 | | 6.02 | C_21_H_19_O_12_ | 463.08820 | 463.08862 | -0.92 | 151.00383(3), 178.99899(3), 271.02512(3), **300.02798**(100), 301.03580(42), 463.08875(4) | Oszmiański et al. 2015 |
| **51** | Kaempferol 3-O-(4"-hexosyl)-rhamnoside (Multiflorin B) | | 6.03 | C_27_H_29_O_15_ | 593.15119 | 593.15203 | -1.41 | 151.00381(4), 255.03055(5), 257.03796(4), **284.03302**(100), 285.03687(94) | Wei et al. 2004 |
| **52** | Kaempferol glycoside derivative 5 | | 6.04 | C_54_H_59_O_29_ | 1171.31475 | 1171.31571 | -0.82 | 255.03015(3), **284.03287**(100), 285.03998(18), 429.08264(4), 431.09805(4), 593.15173(37) | / |
| **53** | Kaempferol glycoside derivative 6 | | 6.04 | C_49_H_57_O_27_ | 1077.30927 | 1077.30990 | -0.58 | 284.03311(69), 285.04077(34), 593.15137(8), 737.19409(14), **755.20563**(100), 931.25189(13) | / |
| **54** | Kaempferol glycoside derivative 7 | | 6.08 | C_48_H_55_O_26_ | 1047.29872 | 1047.30025 | -1.47 | 284.03302(59), 285.04062(24), 593.15179(7), 737.19476(10), **755.20508**(100), 901.24225(10) | / |
| **55** | Isorhamnetin 3-O-(6"-pentosyl)-hexoside | | 6.08 | C_27_H_29_O_16_ | 609.14611 | 609.14685 | -1.21 | 271.02551(4), 299.02032(13), 300.02798(5), **314.04373**(100), 315.05121(21) | / |
| **56** | Quercetin glycoside derivative 2 | | 6.09 | C_42_H_45_O_22_ | 901.24081 | 901.24247 | -1.84 | 300.02792(19), **301.03580**(100), 446.08566(28), 447.09348(44), 609.14697(40), 755.20135(17) | / |
| **57** | Isorhamnetin 3-O-(6"-rhamnosyl)-hexoside | | 6.10 | C_28_H_31_O_16_ | 623.16176 | 623.16263 | -1.40 | 299.02036(10), 300.02808(16), 314.04382(53), **315.05157**(100), 623.16168(6) | Oszmiański et al. 2015 |
| **58** | Kaempferol 3,7-di-O-rhamnoside (Kaempferitrin) | | 6.10 | C_27_H_29_O_14_ | 577.15628 | 577.15729 | -1.75 | 284.03268(4), **285.04083**(100), 430.09085(37), 431.09863(24) | / |
| **59** | Quercetin glycoside derivative 3 | | 6.19 | C_62_H_69_O_33_ | 1341.37266 | 1341.37481 | -1.61 | 300.02786(23), 301.03552(12), 446.08542(20), 447.09268(5), 885.23071(13), 903.24152(43), 1031.27124(11), **1049.28088**(100), 1195.32446(36) | / |
| **60** | Naringenin 7-O-(6"-rhamnosyl)-hexoside | | 6.20 | C_27_H_31_O_14_ | 579.17193 | 579.17265 | -1.24 | 151.00397(22), **271.06168**(100) | / |
| **61** | Kaempferol glycoside derivative 8 | | 6.22 | C_57_H_61_O_29_ | 1209.33041 | 1209.33229 | -1.56 | 284.03302(59), 285.04059(25), 446.08560(39), 447.09207(6), 753.18951(18), **771.20001**(100), 899.23834(7), 917.24396(81), 1063.29053(11) | / |
| **62** | Kaempferol glycoside derivative 9 | | 6.23 | C_63_H_71_O_33_ | 1355.38831 | 1355.38951 | -0.89 | 284.03305(46), 285.04056(22), 917.25751(31), 1045.28333(15), **1063.29419**(100), 1209.33167(56) | / |
| **63** | Kaempferol glycoside derivative 10 | | 6.24 | C_42_H_45_O_21_ | 885.24599 | 885.24720 | -1.36 | 145.02982(6), 284.03311(8), **285.04089**(100), 431.09857(13), 593.15283(5) | / |
| **64** | Kaempferol 3-O-hexoside | | 6.24 | C_21_H_19_O_11_ | 447.09329 | 447.09356 | -0.62 | 151.00389(4), **284.03293**(100), 285.04074(44), 447.09329(43) | Owczarek et al. 2021 |
| **65** | Kaempferol 3-O-(2"-pentosyl)-rhamnoside | | 6.28 | C_26_H_27_O_14_ | 563.14062 | 563.14189 | -2.26 | 255.03014(3), **284.03293**(100), 285.04031(11), 563.14166(7) | / |
| **66** | Kaempferol glycoside derivative 11 | | 6.31 | C_62_H_69_O_32_ | 1325.37774 | 1325.37897 | -0.92 | 284.03281(44), 285.04025(22), 887.24597(30), 1015.27222(14), **1033.28223**(100), 1179.31934(51) | / |
| **67** | Isorhamnetin 3-O-hexoside | | 6.32 | C_22_H_21_O_12_ | 477.10385 | 477.10430 | -0.94 | 243.03053(5), 271.02551(7), **314.04382**(100), 315.05145(11), 477.10474(23) | Oszmiański et al. 2015 |
| **68** | Naringenin 7-O-hexoside | | 6.32 | C_21_H_21_O_10_ | 433.11402 | 433.11429 | -0.61 | 119.05054(5), 151.00398(20), 177.01982(3), **271.06177**(100) | Liu et al. 2023 |
| **69** | Kaempferol glycoside derivative 12 | | 6.33 | C_60_H_59_O_27_ | 1211.32498 | 1211.32723 | -1.85 | 284.03278(28), 285.03970(16), 755.20374(37), 883.23047(12), **901.24042**(100), 1047.27783(50) | / |
| **70** | Kaempferol glycoside derivative 13 | | 6.35 | C_58_H_63_O_29_ | 1223.34605 | 1223.34737 | -1.08 | **284.03317**(100), 285.04065(44), 755.20538(73), 769.21625(8), 901.24194(33), 931.25354(42) | / |
| **71** | Kaempferol 3-O-(6′′-malonyl)-hexoside | | 6.40 | C_24_H_21_O_14_ | 533.09368 | 533.09480 | -2.10 | 255.03014(3), **284.03293**(100), 285.04077(84) | / |
| **72** | Kaempferol 3-O-[6'''-acetyl-(4"-hexosyl)]-rhamnoside (Multiflorin A) | | 6.42 | C_29_H_31_O_16_ | 635.16176 | 635.16303 | -2.00 | 255.03027(7), **284.03287**(100), 285.03888(9) | / |
| **73** | Kaempferol 3-O-(6′′-acetyl)-hexoside | | 6.42 | C_23_H_21_O_12_ | 489.10385 | 489.10462 | -1.58 | 255.03038(5), **284.03305**(100), 285.04083(68) | / |
| **74** | Isorhamnetin 3-O-(6′′-acetyl)-hexoside | | 6.49 | C_24_H_23_O_13_ | 519.11442 | 519.11529 | -1.69 | 299.02017(24), **300.02808**(100), 314.0437(26), 315.05188(31) | / |
| **75** | Quercetin 3-O-hexoside isomer 2 | | 6.49 | C_21_H_19_O_12_ | 463.08820 | 463.08864 | -0.96 | 151.00394(11), 178.99893(10), 300.02777(3), **301.03555**(100) | Oszmiański et al. 2015 |
| **76** | Isorhamnetin 3-O-rhamnoside | | 6.50 | C_22_H_21_O_11_ | 461.10894 | 461.10933 | -0.86 | 283.0253(46), 284.03275(10), 298.04904(46), 299.05661(24), 313.03522(9), **446.08594**(100) | Oszmiański et al. 2015 |
| **77** | Kaempferol 3-O-rhamnoside | | 6.54 | C_21_H_19_O_10_ | 431.09837 | 431.09876 | -0.91 | 255.03091(12), 257.04718(3), **284.03320**(100), 285.04092(90) | Oszmiański et al. 2015 |
| **78** | Apigenin 7-O-hexoside | | 6.71 | C_21_H_19_O_10_ | 431.09837 | 431.09887 | -1.15 | 225.05629(19), **269.04608**(100) | Sarikurkcu et al. 2020 |
| **Flavonoid aglycones** | | | | | | | | | |
| **79** | Eriodictyol | | 5.99 | C_15_H_11_O_6_ | 287.05611 | 287.05659 | -1.65 | **125.02468**(100), 161.02492(35), 219.06679(27), 243.06723(6), 287.05673(9) | Sarikurkcu et al. 2020 |
| **80** | Taxifolin | | 6.01 | C_15_H_11_O_7_ | 303.05103 | 303.05158 | -1.83 | **125.02472**(100), 137.02480(55), 153.01956(11), 175.0406(18), 177.01965(14), 285.04138(32) | Sarikurkcu et al. 2020 |
| **81** | Hesperetin | | 6.41 | C_16_H_13_O_6_ | 301.07176 | 301.07229 | -1.76 | 107.01431(11), 149.06104(33), **151.00394**(100), 164.01224(14), 177.01987(13), 286.04941(11) | / |
| **82** | Quercetin | | 6.98 | C_15_H_9_O_7_ | 301.03538 | 301.03611 | -2.43 | 107.01405(6), 121.02984(17), **151.00406**(100), 178.99908(57), 273.04095(10), 301.03613(83) | Owczarek et al. 2021 |
| **83** | Naringenin | | 7.33 | C_15_H_11_O_5_ | 271.06120 | 271.06183 | -2.32 | 107.01408(9), 119.05048(41), **151.00398**(100), 177.01875(11), 271.06146(48) | Liu et al. 2023 |
| **84** | Apigenin | | 7.36 | C_15_H_9_O_5_ | 269.04555 | 269.04617 | -2.32 | **269.04620**(100) | Sarikurkcu et al. 2020 |
| **85** | Kaempferol | | 7.42 | C_15_H_9_O_6_ | 285.04046 | 285.04110 | -2.26 | 257.04617(2), **285.04080**(100) | Owczarek et al. 2021 |
| **86** | Isorhamnetin | | 7.50 | C_16_H_11_O_7_ | 315.05103 | 315.05193 | -2.85 | **300.02823**(100), 315.05212(52) | Wollenweber and Egger 1970 |
| **Saponins** | | | | | | | | | |
| **87** | Aescin derivative 1 (like escin IV) | | 6.49 | C_52_H_81_O_24_ | 1089.51233 | 1089.51376 | -1.32 | 619.38611(6), 715.4057(6), 927.46063(4), 957.47137(23), **1089.51367**(100) | Wang et al. 2023 |
| **88** | Aesculus saponin 1 | | 6.66 | C_42_H_67_O_17_ | 843.43838 | 843.43886 | -0.58 | 101.02464(9), 113.02464(14), 619.38422(3), 663.37585(8), **843.43866**(100) | Li et al. 2023 |
| **89** | Aesculiside N | | 6.74 | C_49_H_77_O_22_ | 1017.49121 | 1017.49125 | -0.04 | **113.02460**(100), 119.03514(43), 139.00381(38), 157.01471(5), 547.36273(10), 1017.49054(28) | Zhang et al. 2020 |
| **90** | Aesculus saponin 2 | | 7.57 | C_58_H_89_O_26_ | 1201.56476 | 1201.56690 | -1.78 | 157.01436(21), 827.45673(7), 1039.51807(6), 1069.52527(28), 1187.59363(12), **1201.56689**(100) | / |
| **91** | Aesculus saponin 3 | | 7.71 | C_55_H_91_O_29_ | 1215.56522 | 1215.56714 | -1.58 | 157.01456(21), 841.45782(6), 1053.51367(6), 1083.5249(31), **1215.56714**(100) | / |
| **92** | Assamicin VIII | | 7.86 | C_47_H_73_O_18_ | 925.48025 | 925.48164 | -1.51 | 113.02460(34), 119.03513(11), 157.01440(9), 745.4162(7), 825.42902(9), **925.47864**(100) | Wang et al. 2023 |
| **93** | Aesculioside IIc | | 8.03 | C_52_H_81_O_21_ | 1041.52758 | 1041.52815 | -0.55 | 113.02468(29), 157.01471(12), 641.43170(10), 911.49030(23), **1043.53333**(100) | Zhang et al. 2006 |
| **94** | Aescin derivative 2 (like escin IVf) | | 8.04 | C_53_H_83_O_23_ | 1087.53306 | 1087.53453 | -1.35 | 113.02468(52), 119.03533(16), 157.01479(17), 685.43256(21), 955.49316(30), **1087.53357**(100) | Wang et al. 2023 |
| **95** | Aesculioside IIa or IIb | | 8.06 | C_52_H_81_O_22_ | 1057.52250 | 1057.52357 | -1.02 | 119.03523(11), 157.01472(13), 655.44061(16), 925.5022(25), 957.47113(7), **1057.54578**(100) | Zhang et al. 2006 |
| **96** | Aesculus saponin 4 | | 8.11 | C_59_H_89_O_25_ | 1197.56984 | 1197.57031 | -0.39 | 226.33009(4), 1065.52673(18), **1197.57031**(100) | / |
| **97** | Aescin derivative 3 (like escin V) | | 8.22 | C_54_H_85_O_24_ | 1117.54363 | 1117.54410 | -0.42 | **119.03517**(100), 139.00388(99), 143.03520(17), 161.04575(31), 179.05653(18), 617.40607(12) | Wang et al. 2023 |
| **98** | Aescin derivative 4 (like isoescin IIb) | | 8.29 | C_54_H_83_O_23_ | 1099.53317 | 1099.53448 | -1.19 | 119.03522(13), 157.01459(17), 629.40570(7), 631.41449(12), 969.49927(20), **1101.54126**(100) | Wang et al. 2023 |
| **99** | Aesculus saponin 5 | | 8.69 | C_47_H_75_O_17_ | 911.50108 | 911.50195 | -0.95 | 113.02480(17), 157.01462(8), 509.40012(14), 779.45929(22), **911.50195**(100) | / |
| **100** | Aesculus saponin 6 | | 8.38 | C_51_H_81_O21 | 1029.52758 | 1029.52918 | -1.55 | 113.02467(30), 157.01414(13), 457.32968(4), 589.41534(15), 897.48389(22), **1029.52917**(100) | / |
| **101** | Putranoside C | | 8.42 | C_48_H_75_O_18_ | 939.49589 | 939.49609 | -0.22 | 113.02465(52), 119.03513(17), 157.01447(13), 759.43201(11), 789.44257(5), **939.49609**(100) | Wang et al. 2010 |
| **102** | Aesculus saponin 7 | | 8.44 | C_49_H_75_O_19_ | 967.49080 | 967.49190 | -1.13 | 101.02466(29), 113.02465(44), 119.03526(14), 787.42847(8), 805.43988(3), **967.49158**(100) | Li et al. 2023 |
| **103** | Escin derivative 5 (like pavioside D) | | 8.59 | C_54_H_83_O_22_ | 1083.53815 | 1083.53992 | -1.64 | **113.02463**(100), 119.03515(39), 131.03529(11), 139.00391(24), 191.05629(14), 611.39465(10), 613.41107(10), 681.43787(69), 1083.53992(32) | Lanzotti et al. 2012 |
| **104** | Aescin derivative 6 (like isoescin VIIa) | | 8.60 | C_55_H_85_O_24_ | 1129.54363 | 1129.54482 | -1.05 | 119.03565(13), 127.07670(31), 157.01451(23), 727.47943(23), 997.53802(35), **1129.58069**(100) | Wang et al. 2023 |
| **105** | Aesculioside G | | 8.75 | C_56_H_87_O_24_ | 1143.55928 | 1143.56129 | -1.76 | 119.03535(11), 139.00407(10), 157.01471(20), 673.43304(20), 1011.51929(26), **1143.56128**(100) | Li et al. 2023 |
| **106** | Aesculus saponin 8 | | 8.87 | C_52_H_79_O_20_ | 1023.51703 | 1023.51868 | -1.62 | 113.02464(45), 119.03495(12), 157.01442(13), 843.46338(7), 893.45361(7), **1023.51868**(100) | / |
| **107** | Aesculus saponin 9 | | 8.88 | C_51_H_77_O_19_ | 993.50656 | 993.50775 | -1.19 | 113.02469(28), 157.01460(9), 843.45416(6), 861.46411(5), **993.50775**(100) | / |
| **108** | Aesculioside IIIc | | 8.88 | C_56_H_87_O_23_ | 1127.56436 | 1127.56764 | -2.90 | **119.03511**(100), 131.03499(22), 139.00383(62), 149.04593(14), 157.01472(7), 161.04601(10), 629.40454(12), 657.43506(18), 1127.56763(11) | Wang et al. 2010 |
| **109** | Aesculus saponin 10 | | 9.05 | C_59_H_93_O_24_ | 1185.60574 | 1185.60902 | -2.76 | 145.08711(36), 157.01485(36), 226.33412(16), 737.46094(14), 783.50739(23), 1007.52435(17), 1053.56946(37), 1139.57324(51), **1185.60901**(100) | / |
| **110** | Aesculiside P | | 9.06 | C_57_H_87_O_23_ | 1139.56436 | 1139.56542 | -0.93 | 737.46436(11), 977.51184(3), 1007.52271(17), **1139.56494**(100) | Zhang et al. 2020 |
| **111** | Aesculioside IV a or IVb | | 9.08 | C_57_H_89_O_23_ | 1141.58004 | 1141.58014 | -0.08 | 157.01454(7), 739.47913(7), 1009.53613(18), **1141.57983**(100) | Zhang et al. 2006 |
| **112** | Aesculus saponin 3 | | 9.12 | C_52_H_79_O_19_ | 1007.52210 | 1007.52320 | -1.09 | 119.03520(11), 157.01457(11), 161.04559(4), 669.43579(5), 827.45966(7), **1007.52319**(100) | / |
| **113** | Aesculus saponin 11 | | 9.14 | C_56_H_85_O_22_ | 1109.55380 | 1109.55457 | -0.70 | 157.01454(25), 669.43756(30), 977.51239(34), **1109.55457**(100) | / |
| **114** | Aesculus saponin 12 | | 9.29 | C_51_H_79_O_18_ | 979.52719 | 979.52820 | -1.03 | 113.02478(42), 157.01474(10), 671.45367(7), 829.47644(5), **979.52820**(100) | / |
| **115** | Aesculioside IV-23D1 | | 9.38 | C_57_H_87_O_22_ | 1123.56945 | 1123.57084 | -1.24 | 157.01457(21), 651.42719(14), 721.46991(31), 991.52802(41), **1123.57080**(100) | Zhang et al. 2006 |
| **116** | Aesculioside D or C | | 9.40 | C_58_H_89_O_24_ | 1169.57493 | 1169.57676 | -1.57 | 157.01512(10), 161.04657(4), 651.42871(5), 721.47015(18), 991.53082(23), **1123.57117**(100) | Wang et al. 2023 |
| **117** | Aesculus saponin 13 | | 9.66 | C_57_H_87_O_21_ | 1107.57454 | 1107.57593 | -1.26 | 157.01445(22), 635.43317(16), 705.47406(29), 733.46826(9), 975.53320(38), **1107.57593**(100) | / |

**References**

Bielarska, A. M., Jasek, J. W., Kazimierczak, R., and Hallmann, E. (2022). Red horse chestnut and horse chestnut flowers and leaves: a potential and powerful source of polyphenols with high antioxidant capacity. Molecules 27, 2279. [doi.org/10.3390/molecules27072279](https://doi.org/10.3390/molecules27072279)

Deyab, M. A., Mohsen, Q., and Guo, L. (2022). *Aesculus hippocastanum* seeds extract as eco-friendly corrosion inhibitor for desalination plants: Experimental and theoretical studies. *J. Mol. Liq.* 361, 119594. [doi.org/10.1016/j.molliq.2022.119594](https://doi.org/10.1016/j.molliq.2022.119594)

Dias, M. I., Albiston, C., Añibarro-Ortega, M., Ferreira, I. C. F. R., Pinela, J., and Barros, L. (2022). Sonoextraction of phenolic compounds and saponins from *Aesculus hippocastanum* seed kernels: Modeling and optimization. *Ind. Crops Prod.* 185, 115142. [doi.org/10.1016/j.indcrop.2022.115142](https://doi.org/10.1016/j.indcrop.2022.115142)

Dudek-Makuch, M., Studzińska-Sroka, E., Korybalska, K., Czepulis, N., Łuczak, J., Rutkowski, R., et al. (2019). Biological activity of *Aesculus hippocastanum* flower extracts on vascular endothelial cells cultured *in vitro*. *Phytoch. Lett.* 30, 367–375. [doi.org/10.1016/j.phytol.2019.02.031](https://doi.org/10.1016/j.phytol.2019.02.031)

Kapusta, I., Janda, B., Szajwaj, B., Stochmal, A., Piacente, S., Pizza, C., et al. (2007). Flavonoids in horse chestnut (*Aesculus hippocastanum*) seeds and powdered waste water byproducts. *J. Agric. Food Chem.* 55, 8485–8490. [doi.org/10.1021/jf071709t](https://doi.org/10.1021/jf071709t)

Kimura, H., Ogawa, S., Ishihara, T., Maruoka, M., Tokuyama-Nakai, S., Jisaka, M., et al. (2017). Antioxidant activities and structural characterization of flavonol O-glycosides from seeds of Japanese horse chestnut (*Aesculus turbinata* BLUME). *Food Chem.* 228, 348–355. [doi.org/10.1016/j.foodchem.2017.01.084](https://doi.org/10.1016/j.foodchem.2017.01.084)

Lanzotti, V., Termolino, P., Dolci, M., and Curir, P. (2012). Paviosides A–H, eight new oleane type saponins from *Aesculus pavia* with cytotoxic activity. *Bioorg. Med. Chem.* 20, 3280–3286. [doi.org/10.1016/j.bmc.2012.03.048](https://doi.org/10.1016/j.bmc.2012.03.048)

Li, H., Cao, H., Ruan, J., Wu, Y., Yang, D., Gao, Q., et al. (2023). Saponins from *Aesculus wilsonii* seeds exert anti-inflammatory activity through the suppression of NF-κB and NLRP3 pathway. *Arab. J. Chem.* 16, 105077. [doi.org/10.1016/j.arabjc.2023.105077](https://doi.org/10.1016/j.arabjc.2023.105077)

Liu, M., Liu, G., Wang, G., Song, S., Zhang, P., Liu, X., et al. (2023). Identification and functional characterization of *AcMYB113* in anthocyanin metabolism of *Aesculus chinensis* Bunge var. *chinensis* leaves. *Plant Physiol. Biochem.* 199, 107709. [doi.org/10.1016/j.plaphy.2023.107709](https://doi.org/10.1016/j.plaphy.2023.107709)

Morimoto, S., Nonaka, G., and Nishioka, I. (1987). Tannins and related compounds. LIX. Aesculitannins, novel proanthocyanidins with doubly-bonded structures from *Aesculus hippocastanum* L. Chem. Pharm. Bull. 35, 4717–4729. [doi.org/10.1248/cpb.35.4717](https://doi.org/10.1248/cpb.35.4717)

Oszmiański, J., Kolniak-Ostek, J., and Biernat, A. (2015). The content of phenolic compounds in leaf tissues of *Aesculus glabra* and *Aesculus parviflora* Walt. Molecules 20, 2176–2189. [doi.org/10.3390/molecules20022176](https://doi.org/10.3390/molecules20022176)

Owczarek, A., Kołodziejczyk-Czepas, J., Marczuk, P., Siwek, J., Wąsowicz, K., and Olszewska, M. A. (2021). Bioactivity potential of Aesculus hippocastanum L. flower: Phytochemical profile, antiradical capacity and protective effects on human plasma components under oxidative/nitrative stress *in vitro*. Pharmaceuticals 14, 1301. [doi.org/10.3390/ph14121301](https://doi.org/10.3390/ph14121301)

Santos-Buelga, C., Kolodzieij, H., and Treutter, D. (1995). Procyanidin trimers possessing a doubly linked structure from *Aesculus hippocastanum*. *Phytochemistry* 38, 499–504. [doi.org/10.1016/0031-9422(94)00637-9](https://doi.org/10.1016/0031-9422(94)00637-9)

Sarikurkcu, C., Locatelli, M., Tartaglia, A., Ferrone, V., Juszczak, A. M., Ozer, M. S., et al. (2020). Enzyme and biological activities of the water extracts from the plants *Aesculus hippocastanum*, *Olea europaea* and *Hypericum perforatum* that are used as folk remedies in Turkey. Molecules 25, 1202. [doi.org/10.3390/molecules25051202](https://doi.org/10.3390/molecules25051202)

Wang, P., Ownby, S., Zhang, Z., Yuan, W., and Li, S. (2010). Cytotoxicity and inhibition of DNA topoisomerase I of polyhydroxylated triterpenoids and triterpenoid glycosides. *Bioorg. Med. Chem. Lett.* 20, 2790–2796. [doi.org/10.1016/j.bmcl.2010.03.063](https://doi.org/10.1016/j.bmcl.2010.03.063)

Wang, W. W., Liu, Z. W., Sun, Y. Z., and Liu, L. F. (2023). Comparison of semen *Aesculi* species from different geographical origins and horse chestnut based on metabolomics and chemometrics analysis. *Phytochem. Analysis* 34,  830–841. [doi.org/10.1002/pca.3212](https://doi.org/10.1002/pca.3212)

Wei, F., Ma, L. Y., Jin, W. T., Ma, S. C., Han, G. Z., Khan, I. A., et al. (2004). Antiinflammatory triterpenoid saponins from the seeds of *Aesculus chinensis*. *Chem. Pharm. Bull.* 52, 1246–1248.  [doi.org/10.1248/cpb.52.1246](https://doi.org/10.1248/cpb.52.1246)

Wollenweber, E., and Egger, K. (1970). Methyläther des myricetins, quercetins und kämpferols im knospenöl von *Aesculus hippocastanum*. *Tetrahedron Lett.* 11, 1601–1604. [doi.org/10.1016/S0040-4039(01)98034-7](https://doi.org/10.1016/S0040-4039(01)98034-7)

Zhang, Z., Li, S., Zhang, S., and Gorenstein, D. (2006). Triterpenoid saponins from the fruits of Aesculus pavia. *Phytochem.* 67, 784. [doi.org/10.1016/j.phytochem.2006.01.017](https://doi.org/10.1016/j.phytochem.2006.01.017)

Zhang, N., Wei, S., Cao, S., Zhang, Q., Kang, N., Ding, L., et al. (2020). Bioactive triterpenoid saponins from the seeds of *Aesculus chinensis* Bge. var. *chekiangensis*. *Front. Chem.* 7, 908. [doi.org/10.3389/fchem.2019.00908](https://doi.org/10.3389/fchem.2019.00908)
